# Biochemical Behavior, Influence on Cell DNA Condition, and Microbiological Properties of Wool and Wool–Copper Materials

**DOI:** 10.3390/ma17122878

**Published:** 2024-06-12

**Authors:** Zdzisława Mrozińska, Anna Kaczmarek, Małgorzata Świerczyńska, Michał Juszczak, Marcin H. Kudzin

**Affiliations:** 1 Łukasiewicz Research Network—Lodz Institute of Technology, 19/27 Marii Sklodowskiej-Curie Str., 90-570 Lodz, Poland; zdzislawa.mrozinska@lit.lukasiewicz.gov.pl (Z.M.); michal.juszczak@lit.lukasiewicz.gov.pl (M.J.); 2 Institute of Polymer and Dye Technology, Faculty of Chemistry, Lodz University of Technology, Stefanowskiego 16, 90-537 Lodz, Poland; 3 Department of Molecular Genetics, Faculty of Biology and Environmental Protection, University of Lodz, 90-236 Lodz, Poland

**Keywords:** antifungal activity, antimicrobial activity, blood coagulation, DNA damage, activated partial thromboplastin time, magnetron sputtering, melt-blown, wool, plasmid relaxation assay, pro-thrombin time, copper

## Abstract

The paper presents the study concerning the preparation and physio-chemical and biological properties of wool–copper (WO-Cu) materials obtained by the sputter deposition of copper onto the wool fibers. The WO-Cu material was subjected to physio-chemical and biological investigations. The physio-chemical investigations included the elemental analysis of materials (C, N, O, S, and Cu), their microscopic analysis, and surface properties analysis (specific surface area and total pore volume). The biological investigations consisted of the antimicrobial activity tests of the WO-Cu materials against colonies of Gram-positive (*Staphylococcus aureus*) bacteria, Gram-negative (*Escherichia coli*) bacteria, and fungal mold species (*Chaetomium globosum*). Biochemical–hematological tests included the evaluation of the activated partial thromboplastin time and pro-thrombin time. The tested wool–copper demonstrated the ability to interact with the DNA in a time-dependent manner. These interactions led to the DNA’s breaking and degradation. The antimicrobial and antifungal activities of the WO-Cu materials suggest a potential application as an antibacterial/antifungal material. Wool–copper materials may be also used as customized materials where the blood coagulation process could be well controlled through the appropriate copper content.

## 1. Introduction

Bleeding is a major cause of mortality and morbidity following both military and civilian trauma [1]. Since bleeding is simultaneously accompanied by bacteria invasion, hemostatic and antibacterial dressings may offer effective control as a part of the prehospital treatment.

Subsequent wound healing of the skin [2] presents a very complex biological process [3,4,5], and thus, its effective treatment has become one of the most important challenges for healthcare [6,7,8,9,10,11]. Therefore, various wound dressings have been tested [12,13,14,15,16], among them polymer composites exhibiting effective hemostatic [17,18,19] and antibacterial properties [20,21,22,23,24,25,26].

As the continuation of our research program directed towards antibacterial polymer–metal materials [27,28,29,30,31,32], we present here the study concerning the wool–copper (WO-Cu) material consisting of the keratin matrix [33,34,35] and copper—the transition element existing mainly in three oxidation states (0, +1, and +2) [36], with positive redox potentials (Cu^+^/Cu = 0.52 V; Cu^2+^/Cu = 0.38 V; Cu^2+^/Cu^+^ = 0.52 V) [37] and coordination numbers 2–4 [36], exhibiting a rich coordination chemistry [38,39,40] and a broad spectrum of diverse biological activities.

Copper’s therapeutic spectrum comprises by antipathogenic (antibacterial [41,42,43,44,45,46,47,48,49,50], antiviral [51,52,53,54,55,56,57,58,59,60], and antifungal [61,62,63,64,65,66,67,68,69,70] activity), anticancer [71,72,73,74,75,76,77,78,79,80], hemostatic [81,82,83,84,85,86,87,88,89,90], angiogenic [91,92,93,94,95,96,97,98,99,100], and osteogenic [96,101,102,103,104,105,106,107,108,109] applications.

In spite of the great medical potential of both components of the WO-Cu material, only a few reports concerning the biochemical investigations of this combination of materials have been published [110,111].

The tested WO-Cu materials were prepared using DC magnetron sputtering to deposit copper on the wool fabrics. The WO-Cu samples were characterized by a complex of physio-chemical and biological/biochemical tests. The main biological aim of the current study is to test the major hemolysis parameters of the wool–copper materials, namely activated partial thromboplastin time (APTT) and pro-thrombin time (PT) [112]. Plasmid relaxation assay is a common method to determine the potential of the tested probe to directly interact with the DNA. As a result of these interactions, it is possible to induce DNA breaks, resulting in a change in an electrophoretic mobility. Herein, we analyzed the potential of wool fibers and wool–copper materials to directly interact with the plasmid DNA.

## 2. Materials and Methods

**Figure 1:**
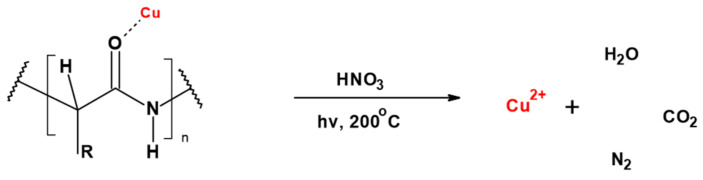
Degradation of the WO-Cu material (hypothetic structure).

**Figure 2:**
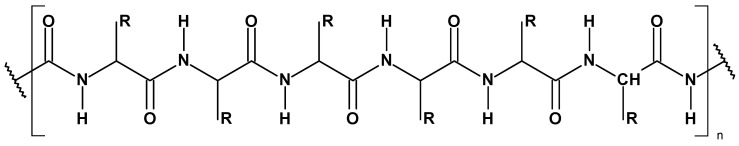
The structure of the keratin chain (R (AA) = H (Gly); Me (Ala); iPr (Val); iBu (Ile); sBu (Leu); Bz (Phe); HO-Bz (Tyr); indolyl (Trp); imidozoylo-CH_2_ (His); hydroxymethyl (Ser); 2-hydroxypropyl (Thr); mercaptomethyl (Cys); methylthiomethyl (Met); 4-aminobutyl (Lys); 4-guanidylobutyl (Arg); carboxymethyl (Asp); carboxamidomethyl (Asn); carboxyethyl (Glu); carboxamidoethyl (Gln)).

**Figure 3:**
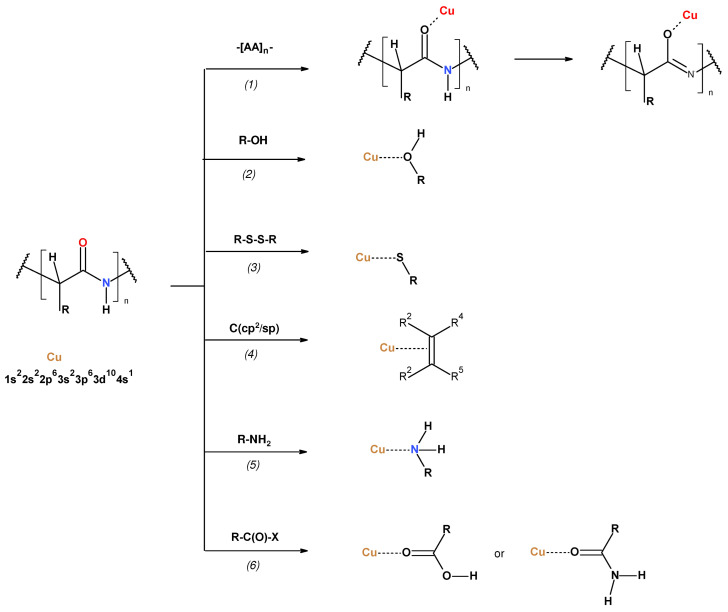
Hypothetic interaction of copper with representative wool functional groups: *(1)* peptide chain; *(2)* hydroxyl functions (Ser, Tyr); *(3)* sulfydryl/ disulfide functions (Cys); *(4)* aryl moieties (Phe, Tyr, His); *(5)* amino/guanidine functions (Lys, Arg); *(6)* carboxyl/carboxamide (Asp, Asn, Glu, Gln). The detailed structures/bonds of AA-Cu adducts are presented in the original papers listed in Supplementary Materials Table S1.

**Figure 4:**
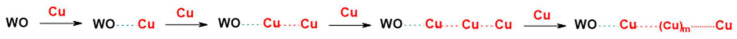
Subsequent putative deposition of copper layers on wool surface (WO → WO∙∙Cu → WO∙∙Cu∙ (Cu)m → WO∙∙Cu∙ (Cu)m∙∙Cu).

All edited figures (Figures 1–4 and 9) in the manuscript were obtained using the BIOVIA Draw 2017 for Academics program.

### 2.1. Materials

#### 2.1.1. Materials Used for Fabrication

The following materials were used to fabricate the investigated WO-Cu samples:

- Copper target from Testbourne Ltd. (Basingstoke, UK) with 99.99% purity;
- Woolen-adjacent fabric, manufactured by SDL ATLAS Textile Testing Solutions (Rock Hill, SC, USA). The characteristics of the woolen-adjacent fabric are presented in Table 1.

#### 2.1.2. Microbiological Strains Used for Antimicrobial Activity Assesment

The following bacterial and fungal strains were purchased from Microbiologics (St. Cloud, MN, USA):

- *Escherichia coli* (ATCC 25922);
- *Staphylococcus aureus* (ATCC 6538);
- *Chaetomium globosum* (ATCC 6205).

### 2.2. Methods

#### 2.2.1. Magnetron Sputtering Modification

The wool underwent copper coating using the magnetron sputtering method. The sputtering procedure parameters are presented in Table 2.

#### 2.2.2. Wool—Copper Material Physio-Chemical Characterization

##### Atomic Absorption Spectrometry with Flame Excitation (FAAS)

The copper content of WO-Cu materials was assessed by prior sample mineralization (Figure 1), using single-module Magnum II microwave mineralizer from Ertec (Wroclaw, Poland) and subsequent FAAS determination, in a similar way as described earlier [27,28,29,30,31,32].

The FAAS determinations were performed using flame excitation with a Thermo Scientific Thermo Solar M6 (LabWrench, Midland, ON, Canada). The spectrometer was equipped with a 100 mm titanium burner, coded lamps with a single-element hollow cathode, background correction: D2 deuterium lamp.

The following Equation (1) [117] was used in order to calculate the total copper content (bulk copper content) in the WO-Cu material:

(1) $$M=\frac{C\times V}{m}$$

where

*M*—total copper content (bulk copper content) (mg/kg);
*C*—metal concentration in the tested solution (mg/L);
*V*—volume of the sample solution (mL);
*m*—mass of the mineralized sample (g).

In order to evaluate the durability/washing fastness of the copper coating, the cooper concentration in WO-Cu materials after washing was determined using the FAAS method and compared with the copper concentration in the same sample before the washing procedure. The washing of the functionalized wool fabrics was performed in accordance with the EN ISO 105-C06:2010 standard [118]. For that purpose, a standard detergent was applied with a concentration equal to 4 g/L. The washing was carried out for 30 min at 40 °C.

##### Microscopic Analysis

The assessment of the surface morphology of the tested samples was carried out using the optical and scanning electron microscopy.

Optical microscopy investigations were performed with the use of a DM6 M microscope (Leica, Wetzlar, Germany). The applied magnification levels were equal to 150× and 2000×.

The SEM (scanning electron microscope) analysis of the WO-Cu materials was carried out with the Phenom ProX G6 scanning electron microscope (Thermo Fisher Scientific, Waltham, MA, USA). The SEM microscopic analysis was performed under low vacuum (60 Pa), and the energy of the probe beam was equal to 15 ekV. The back-scattered electron detector was used. The applied magnifications were 1000× and 8000×.

The performance of an EDS system was evaluated by measuring the resolution of a known set of elemental standards (Oxford Instruments, Abingdon, UK) in line with the ISO 15632:2012 [119].

##### Specific Surface Area and Total Pore Volume Analysis

In order to assess the specific surface area and total pore volume, the Brunauer–Emmet–Teller method (BET) was applied. The Autosorb-1 apparatus (Quantachrome Instruments, Boynton Beach, FL, USA) was used for the measurements with nitrogen (−195.8 °C) applied as a sorption agent. Before the analysis, the samples were dried for 24 h at 105 °C. After that, the samples were degassed at room temperature. For each measurement, ~2 g of a given sample was weighed and used. 

#### 2.2.3. Wool–Copper Material Biological Characterization

##### Antimicrobial Properties

The antibacterial and antifungal activity of WO-Cu materials was evaluated according to the EN ISO 20645:2006 [120] and EN 14119: 2003 [121] standards, respectively. The test were performed against *E. coli*, *S. aureus* (tryptic soya agar (TSA) was used), and *C. globosum* (complete mineral salt agar with glucose was used), analogously to our previous works [27,28,29,30,31,32]. The following concentrations of inoculum were used: *E. coli* = 1.3 × 10^8^ CFU/mL; *S. aureus* = 1.9 × 10^8^ CFU/mL; *C. globosum* = 2.5 × 10^6^ CFU/mL. The samples were 30 mm in diameter and were incubated for 24 h at 37 °C in the case of the *E. coli* and *S. aureus* and for 14 days at 29 °C in the case of the *C. globosum* (in accordance with the above-mentioned ISO standards).

##### Biochemical Properties

1. Plasmid relaxation assay

The plasmid relaxation assay was performed similarly to the procedure of Juszczak et al. [122]. The pUC19 plasmid was isolated from the DH5α *E. coli* cells with Isolate II Plasmid Mini Kit (Meridian Bioscience, OH, USA) according to the manufacturer’s instruction. The isolated plasmid quantity and quality were determined by the A260/A280 ratio and gel electrophoresis, respectively. The native form of the pUC19 exists mainly in the supercoiled form (CCC), which is characterized by a relatively high electrophoretic mobility. The plasmid was digested with the restrictase PstI (New England Biolabs, Ipswich, MA, USA) to induce linear (L) form. Topological differences between the CCC and L forms of the plasmid account for their different electrophoretic mobility. The plasmid at 50 ng μL^−1^ was incubated for 2 h and 24 h with WO and WO-Cu samples. Then, the samples were subjected to 1% agarose gel electrophoresis with ethidium bromide staining, visualization under the UV light (302 nm), scanning by a CCD camera, and analysis with the GeneTools 4.3.9.0 by Syngene (Cambridge, UK) software. During the electrophoresis, we also separated 4 μL of 1 kb DNA ladder (GeneRuler 1 kb DNA Ladder, Thermo Scientific, Waltham, MA, USA).

2. Activated Partial Thromboplastin Time (aPTT) and Pro-thrombin Time (PT)

The standard human blood plasma lyophilizates, i.e., Dia-CONT I (Diagon Kft, Budapest, Hungary), were dissolved in 1 mL of a deionized water. A square piece of each sample (1 mg) was added to 200 µL of plasma, vortexed, and incubated for 15 min at 37 °C. For the aPTT measurements, the Dia-PTT (kaolin and cephalin) reagent (Diagon Kft, Budapest, Hungary) was resolved and 0.025 M CaCl_2_ solution reagent (Diagon Kft, Budapest, Hungary) prepared according to the manufacturer’s instruction. The aPTT measurements were performed using a K-3002 OPTIC coagulometer (KSELMED^®^, Grudziadz, Poland). For each sample, 50 µL of plasma sample and 50 µL of suspension of Dia-PTT were introduced into a measuring cuvette and placed in the thermostat of the coagulometer at 37 °C. The mixture was left for 3 min; then, the measurement was started by adding 50 µL of 0.025 M CaCl_2_ solution to the cuvette. 

For the PT assessment, cuvettes with 100 µL of plasma sample were incubated at 37 °C in the thermostat of the coagulometer for 2 min. Next, 100 µL of Dia-PT (Diagon Kft, Budapest, Hungary) was added, and the measurement was started. Dia-PT contained tissue thromboplastin from rabbit brain, calcium ions, and preservative and was shaken each time before adding in order to obtain a homogeneous suspension.

## 3. Results and Discussion

### 3.1. Magnetron Sputtering Modification 

The wool samples were modified by the surface deposition of metallic copper using a direct current (DC) magnetron sputtering system. Wool fibers consisted of 95–98% proteins (about 80–85% keratin), lipids (0.1%), and minerals (0.5%) [34,123,124,125]. The structure of the keratin chain is given in Figure 2. 

The average amino acidic content in wool is presented in Table 3 [124,125,126].

The copper electron configuration of 1s2 2s2 2p6 3s2 3p6 3d10 4s1 4p0p 0p 0 enables its electro-donor as well electro-acceptor reactivity [36]. These reactivities of metallic copper were summarized and illustrated recently [32]. Physio-chemical investigations on the chemisorption of amino acids and peptide on copper phases have been the subject of numerous reports [127,128,129,130,131,132,133,134,135,136,137,138,139,140,141,142,143,144,145,146,147,148,149,150,151,152,153,154,155,156,157,158,159,160,161,162,163,164,165]. Their summed information is listed in Supplemental Information Table S1 and presented schematically in Figure 3.

The subsequent deposition of further copper layers on the wool surface–copper monolayer resulted in the supposed formation of Cu-Cu bonds [166,167,168,169,170,171,172,173] and is presented in Figure 4.

### 3.2. Physico-Chemical Characteristic of WO-Cu Materials 

#### 3.2.1. Atomic Absorption Spectrometry with Flame Excitation (FAAS) 

The evaluation of the copper bulk concentration in wool–copper materials was performed with the use of the FAAS method and is presented in Table 4.

The copper bulk content in the WO-Cu samples depends on the applied sputtering deposition time, and the correlation bewteen the copper bulk content and the deposition time is almost linear: 5 min process—3510 mg/kg (WO-Cu^(5)^(0.06)); 10 min process—9020 mg/kg (WO-Cu^(10)^(0.14)); 15 min process—24,270 mg/kg (WO-Cu^(15)^(0.38)). In addition, the distribution of copper in a WO-Cu material’s bulk after the magnetron sputtering process is uniform.

The copper bulk concentration in the investigated samples after the performed washing (in accordance with the EN ISO 105-C06:2010 standard [118]) is lower; however, the cooper coatings are still present on the surface of the wool fabric. For the WO-Cu^(5)^(0.06) sample, the copper bulk content lowered to ~85% of the original value, for the WO-Cu^(10)^(0.14) to ~88% of the initial bulk concentration, and for the sample WO-Cu^(15)^(0.38) to ~86% of the copper bulk content prior to the washing. Therefore, it may be concluded that the functionalized samples exhibit a satisfactory washing fastness.

#### 3.2.2. Microscopic Analysis

**Figure 5:**
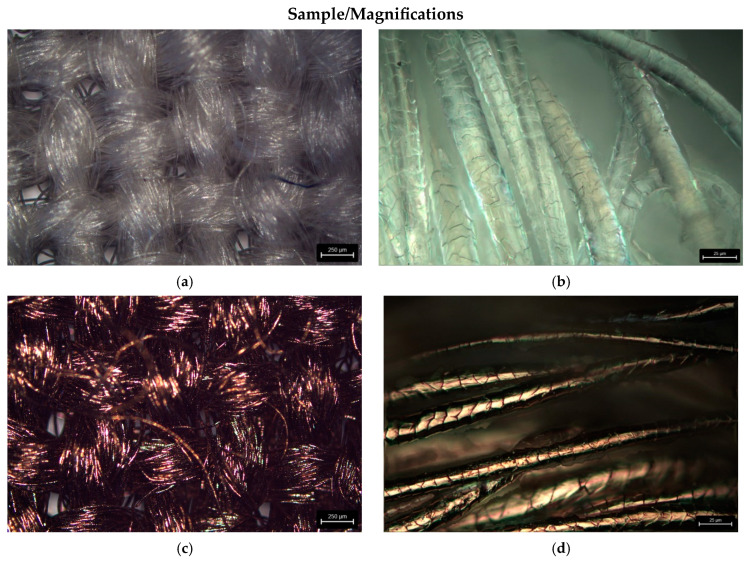
Optical microscopy images (magnifications: ×150 (**a**,**c**); ×2000 (**b**,**d**)) of surface structure of the unmodified wool fabric (**a**,**b**) and WO-Cu^(10)^(0.14) material (**c**,**d**). The scale bar is equal to 250 µm (**a**,**c**) and 25 µm (**b**,**d**).

Figure 5 presents the selected images of the surface morphology before (a,b) and after (c,d) the modification process, obtained using optical microscopy under different magnification levels. It can be clearly observed that the sample was successfully coated by copper (change in the sample color). The copper coating is present not only on the upper fibers of the fabric (placed on the surface) but also on the lower fibers (placed deeper within the fabric). Moreover, the coating is uniform; however, some small spot defects are visible under higher magnification. This may be caused by the presence of some impurities on the surface of the fibers.

**Figure 6:**
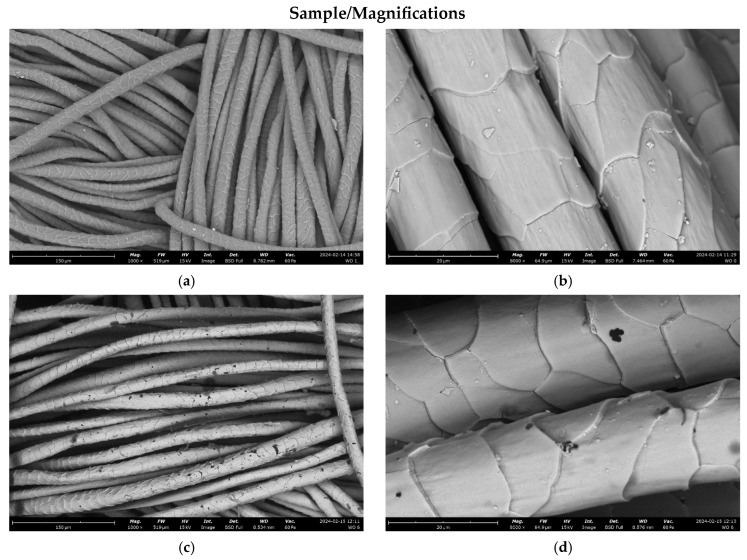
SEM results (magnifications: ×1000 (**a**,**c**); ×8000 (**b**,**d**)) of the tested samples recorded before (WO (**a**,**b**)) and after magnetron sputtering with a copper target (WO-Cu^(10)^(0.14) (**c**,**d**)).

Figure 6 shows the SEM images of the surface of the selected samples before (a,b) and after (c,d) the deposition of copper under different magnifications. It can be observed that both the upper and lower wool fibers are uniformly coated with copper. However, after the magnetron sputtering process, some spot defects are present within the coating (visible as black spots on the surface of the fibers). This is consistent with the results from the optical microscopy.

Both the optical microscopy (Figure 5b) and SEM (Figure 6a,b) observations revealed that the surface of the wool fibers before the magnetron sputtering process consisted of a network of overlapping scales. The untreated wool fibers exhibited a coronal-reticulate pattern of scales [174]. Scales are not only responsible for the characteristic morphological structure of the wool fibers but also play an important role by influencing their properties and by protecting them from damage [175]. From the performed microscopic observation, it may be concluded that the scales remained clearly visible after the magnetron sputter deposition of copper, and their pattern was not altered (Figure 5d and Figure 6c,d). However, the SEM images (Figure 6b,d) indicate that the surface of the scales is smoother after the copper deposition.

**Figure 7:**
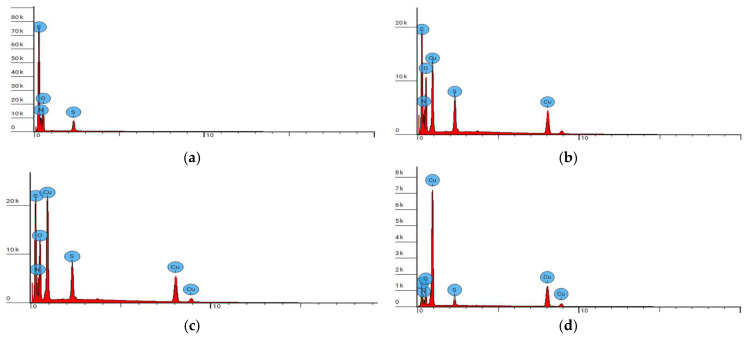
EDS spot analysis diagrams of WO sample and WO-Cu samples: (**a**) WO sample, (**b**) WO-Cu^(5)^(0.06); (**c**) WO-Cu^(10)^(0.14), and (**d**) WO-Cu^(15)^(0.38).

Figure 7 presents the EDS spectra acquired for all of the tested samples. The results of EDS analysis of the chemical composition of the WO sample and WO-Cu samples are listed in Table 5.

In the case of the unmodified sample (WO), the elemental analysis showed the presence of carbon, nitrogen, oxygen, and sulfur, which are the main natural constituents of wool [176]. For the samples modified by the sputter deposition of copper, i.e., WO-Cu^(5)^(0.06), WO-Cu^(10)^(0.14), and WO-Cu^(15)^(0.38), an additional peak corresponding to copper was present.

The relative content of copper increases with the increasing sputter deposition time, which is consistent with the results obtained from the atomic absorption spectrometry. The EDS results confirmed the presence of copper coating on the surface of the wool fabric, and the growing relative content of copper is probably due to the higher thickness of the coating, which is in turn caused by the longer deposition time.

#### 3.2.3. Specific Surface Area and Total Pore Volume Analysis

Table 6 presents the results of the BET analysis of the untreated wool fabric (sample WO) and wool–copper (WO-Cu) materials (WO-Cu^(SpT)^ (MBC)). It was revealed that the specific surface area of the wool fabric was lowered significantly from 0.2653 m^2^/g to 0.1783–0.1929 m^2^/g as a result of the magnetron sputtering deposition of copper. Similarly, the total pore volume also dropped from 7.527 × 10^−4^ cm^3^/g (for the unmodified wool) to 7.103–7.394 × 10^−4^ cm^3^/g (for the samples coated with copper). The observed effect is probably due to the decrease in the porosity of the wool fibers, which results from the fact that the deposited copper filled in the pores present on the fibers’ surface. This is also in agreement with the SEM observations, which showed that the surface of the copper-coated wool fibers is smother than the surface of the untreated wool fibers (Figure 5). The higher deposition time, i.e., the thicker the copper coating, the lower the specific surface area and the lower the total pore volume. 

**Figure 8:**
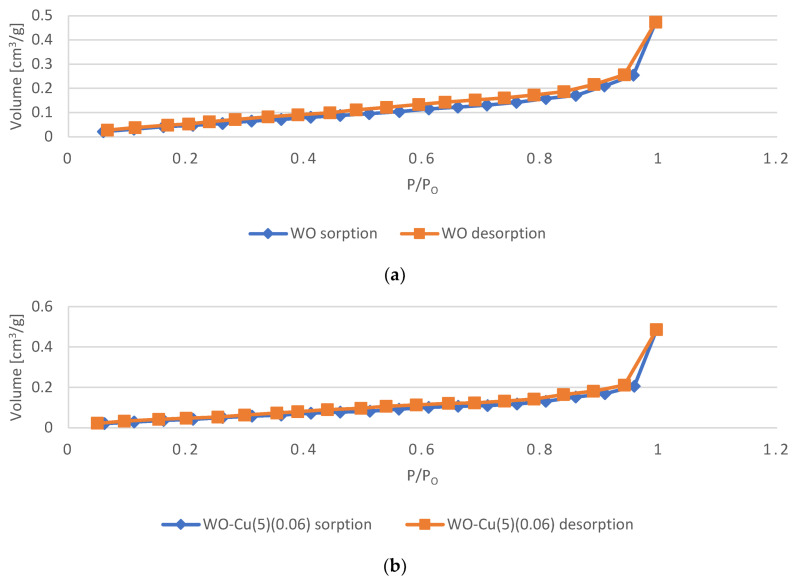

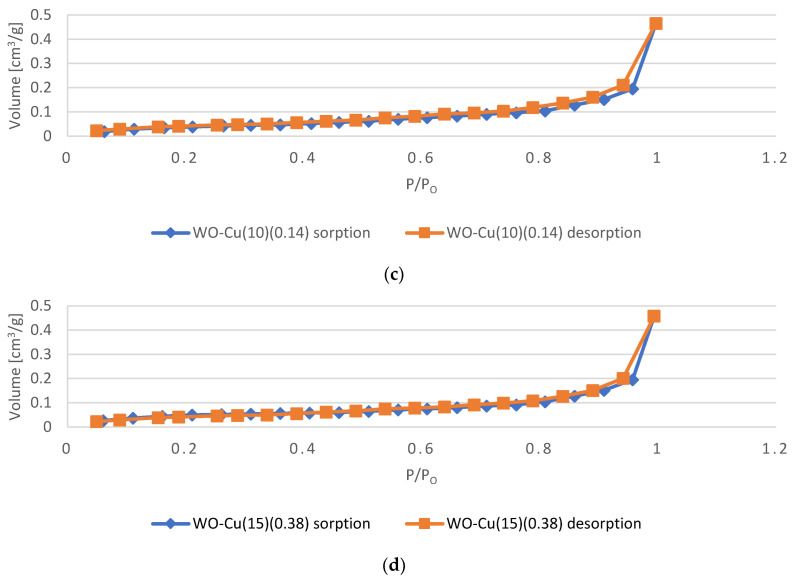
The N_2_ sorption–desorption isotherms obtained for the investigated samples: (**a**) WO; (**b**) WO-Cu^(5)^(0.06); (**c**) WO-Cu^(10)^(0.14); (**d**) WO-Cu^(15)^(0.38).

The N_2_ adsorption–desorption isotherms acquired for the unmodified WO sample and WO-Cu^(SpT)^ (MBC) WO-Cu materials are presented in Figure 8. As it may be observed, the adsorption branch of the isotherm resembles the type II isotherm, which is S-shaped/sigmoid-shaped, according to the International Union of Pure and Applied Chemistry (IUPAC) classification [177,178,179]. This type of isotherms occurs in the case of the physisorption of gases on non- or mesoporous adsorbents and is associated with the monolayer formation followed by the multilayer adsorption [177,178,179]. Since for all of the samples, the observed point B, i.e., the so-called “knee” defined as the beginning of the middle almost linear section, is not so distinctive (a more gradual curvature occurs), it may be concluded that the monolayer formation is overlapped by the multilayer adsorption [178]. It may be observed that with increasing relative pressure, the multilayer adsorption takes place, resulting in an increasing isotherm slope [177]. For all samples, the appearance of the H3 hysteresis loop was observed. The occurrence of the hysteresis loop is a result of the capillary condensation associated with the presence of mesopores [178,179], and the H3 hysteresis loop is related to the slit-shaped pores [178,180].

### 3.3. Microbiological Properties

**Figure 9:**
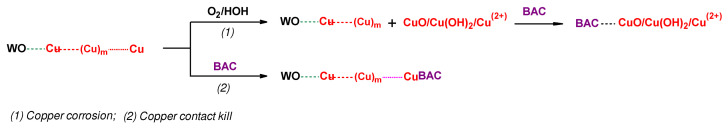
The provisional mechanisms of antibacterial activity of WO-Cu: (1)—corrosion of copper surface with release of Cu^(2+)^ derivatives (according to [181,182]; (2)—contact killing of bacteria [43,45,58,181,182,183,184].

The antimicrobial properties of WO-Cu^(t)^(MBC) materials were investigated by the disk diffusion method, using Gram-negative (*E. coli*) and Gram-positive (*S. aureus*) bacteria and the representative fungus species (*C. globosum*) according to the EN ISO 20645:2006 [120] and EN 14119:2003 [121] standards. Microbiological test results are presented in Table 7, and the images are shown in Figure 9.

**Figure 10:**
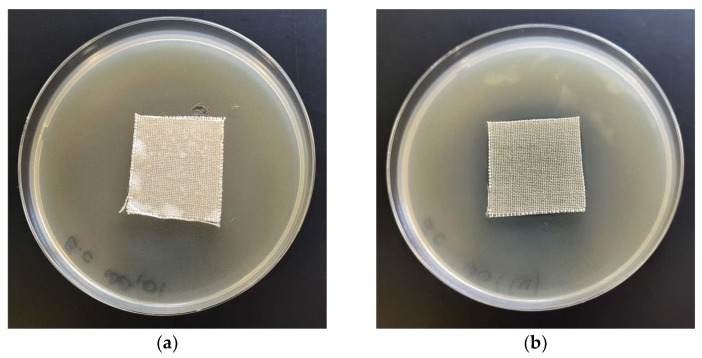

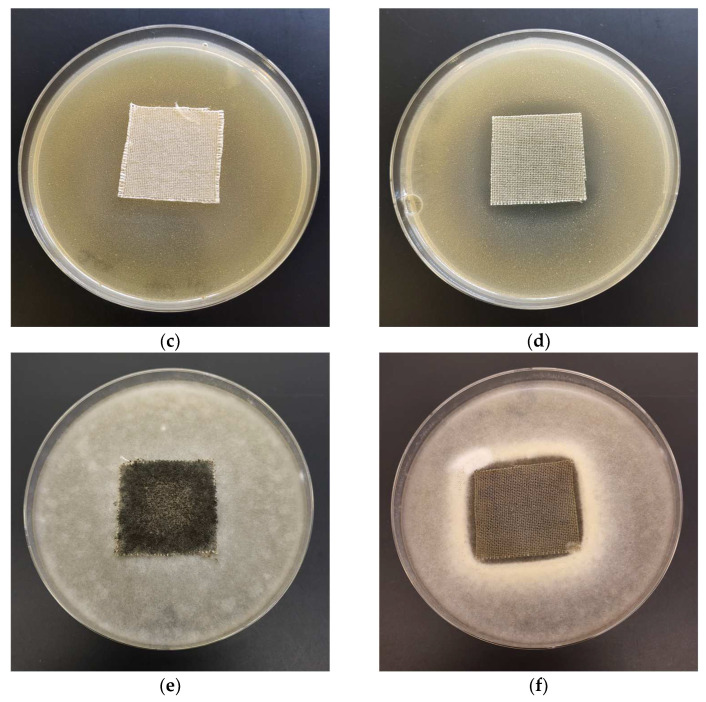
Tests of antimicrobial activity of unmodified wool fabric (**a**,**c**,**e**) and WO-Cu^(15)^(0.38) material (**b**,**d**,**f**) against *E. coli* (**a**,**b**), *S. aureus* (**c**,**d**), and *C. globosum* (**e**,**f**), inhibition zones of bacterial/fungal growth in Petri dishes.

The unmodified material (control/WO) exhibited strong growth of bacterial and fungal colonies, covering the entire surface of the samples placed on the Petri dishes (Figure 10a,c–e; Table 7). WO-Cu^(SpTt)^(MBC) materials showed a good inhibitory effect against the *E. coli* and *S. aureus* bacteria and fungus species (*C. globosum*), expressed by the zones of inhibition (from 1 to 3 mm) and no visible growth on/under the samples (Figure 10b,d,f; Table 7). This assessment was made based on the criteria of the antimicrobial effect according to the EN ISO 20645:2006 [120]. The results obtained in accordance with the EN ISO 20645:2006 and EN 14119:2003 standards [120,121] confirmed the antimicrobial protection ability of the wool–copper (WO-Cu) materials (WO-Cu^(SpT)^(MBC)) against various, representative types of microorganisms. 

The average ZOI of the investigated copper-plated polymers (WO-Cu (this work), PET-Cu [27], and PLA-Cu [28]) (Table 7) are in the range of 1–4 mm due to the low solubility of copper in water [36] and PLA-ALG-Cu^(+2)^ due to formation of strong complexation of cupper ions by alginate [29].

The antibacterial activity of WO-Cu can be caused by released copper ions formed during the surface copper corrosion (Figure 9, path (1)) or copper-contact kill—the interaction of copper’s surface with bacteria membrane (Figure 9, path (2)). 

Table 8 contains the comparison of the representative literature data on diffusion disc assays (ZOI; mm) of copper derivatives and copper nanoparticles. 

These are higher ZOI data compared to those of POLYM-Cu due to the substantially greater solubility of copper ions, derivatives, and nanoparticles in aqueous media (e.g., [36,184,192]).

### 3.4. Biochemical Properties

#### 3.4.1. Plasmid Relaxation Assay

**Figure 11:**
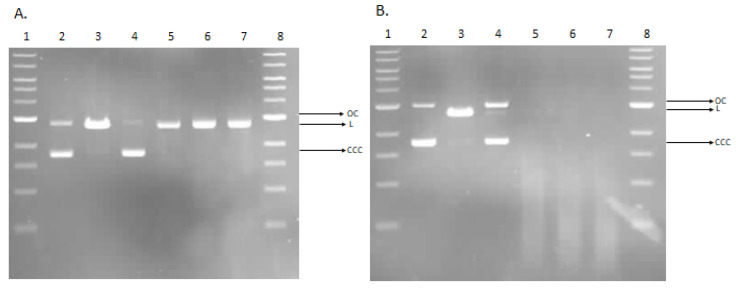
Plasmid relaxation assay. pUC19 plasmid was incubated for 2 h (**A**) and 24 h (**B**) (37 °C) with wool fiber (WO) and wool–copper (WO-Cu) materials WO-Cu^(5)^(0.06), WO-Cu^(10)^(0.14), and WO-Cu^(15)^(0.38) and then was resolved on a 1% agarose gel, stained with ethidium bromide, and visualized in UV light. Line 1—DNA ladder; line 2—pUC19 plasmid (the supercoiled form, CCC); line 3—pUC19 plasmid incubated with restrictase PstI (the linear form, L); lines 4–7—pUC19 plasmid incubated with WO, WO-Cu^(5)^(0.06), WO-Cu^(10)^(0.14), and WO-Cu^(15)^(0.38), respectively; line 8—DNA ladder. OC, the open circular form of pUC19 plasmid.

We investigated the possibility for direct interaction of wool fabric and wool–copper (WO-Cu) materials with the DNA. For this purpose, we used the plasmid relaxation assay. The results obtained from the electrophoretic mobility shift analysis (EMSA) showed that the pUC19 plasmid, which we isolated from the DH5α *E. coli* cells, is presented in the supercoiled form (CCC). Overnight treatment at 37 °C with the restrictase *Pst*I led to a linear form (L) of the plasmid. Incubation of the plasmid with the WO-Cu^(5)^(0.06), WO-Cu^(10)^(0.14), and WO-Cu^(15)^(0.38) samples showed a possibility of DNA adducts or breaks, which affected topological changes of the plasmid and led to the appearance of the OC form, whereas incubation with the WO sample did not affect the plasmid conformation, and the observed results were similar to the control (Figure 11A). This result demonstrates the possibility of the induction of the DNA single-strand breaks by the wool threads in vitro. After longer incubation (24 h), the L form of the plasmid also appeared for the WO sample. In the case of the WO-Cu^(5)^(0.06), WO-Cu^(10)^(0.14), and WO-Cu^(15)^(0.38) samples, we observed smears indicating significant DNA degradation (Figure 11B).

Copper was found as one of the first antimicrobial agents in history. In ancient Egypt, copper solutions were used to clean wounds and to purify water [193]. Studies indicate that copper, especially Cu^2+^, has the ability to interact with DNA [194,195]. Moreover, copper ions can induce Fenton-like reactions and geneate reactive oxygen species (ROS), especially highly reactive hydroxyl radicals [196]. Our results suggest the potential of wool–copper (WO-Cu) materials to directly interact with DNA. These findings could be associated with the antimocrobial activity against the tested species. However, it should be emphasized that the ability to interact with DNA does not imply genotoxic activity for humans. Even if copper from the WO-Cu materials had the opportunity to interact with the genomic DNA, humans have effective DNA repair systems, such as the base excision repair (BER), which remove DNA damage and maintain genome stability [197]. Additionally, the antioxidant systems present in cells protect them from reactive oxygen species [198].

#### 3.4.2. Activated Partial Thromboplastin Time (aPTT) and Pro-Thrombin Time (PTT)

When a foreign body comes into contact with blood, plasma proteins are adsorbed on its surface, which leads to the initiation of the coagulation cascade through the activation of the coagulation factors and the adhesion and activation of platelets. As a result, a fibrin network is formed [199,200,201,202].

Over the last decades, the carboxyl group (-COOH) and the hydroxyl group (-OH) have found a wide application in the design and production of materials characterized by a high compatibility with blood. Carboxyl groups have the ability to bind calcium ions (Ca^2+^) present in the blood, contributing to the improvement of their antithrombotic properties. However, the presence of hydroxyl groups on the materials’ surface promotes their excellent hydrophilicity and leads to antibiofilm properties, which may contribute to the inhibition of the thrombogenesis process by limiting the formation of thrombi [203,204].

**Figure 12:**
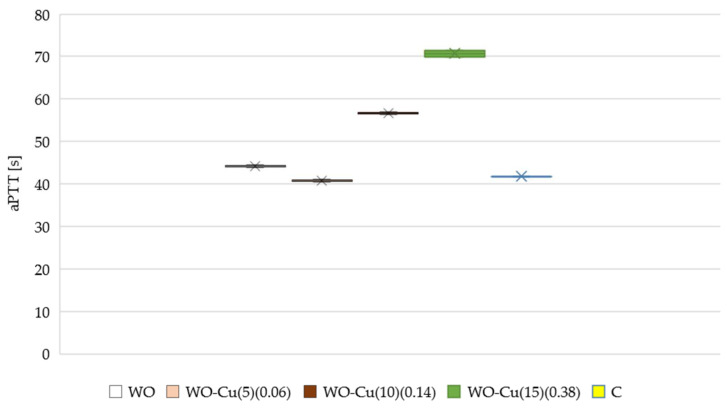
Effect of WO-Cu^(SpT)^(MBC) materials on activated partial thromboplastin time (aPTT). The samples: C, plasma control; WO-Cu^(5)^(0.06); WO-Cu^(10)^(0.14); and WO-Cu^(15)^(0.38). The results are presented as mean (×), median (horizontal line), range (bars), and interquartile range (box).

**Figure 13:**
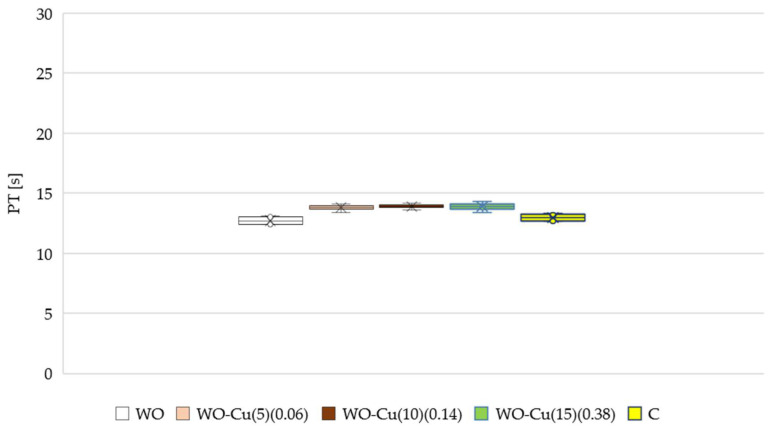
Effect of the WO-Cu^(SpT)^(MBC) materials on pro-thrombin time (PTT). The samples: C, plasma control; WO-Cu^(5)^(0.06); WO-Cu^(10)^(0.14); and WO-Cu^(15)^(0.38). The results are presented as mean (×), median (horizontal line), range (bars), and interquartile range (box).

The blood compatibility of wool materials containing keratin is crucial, and therefore, the assessment of the blood coagulation properties is very important in the context of their use in the medical sector. Activation partial thromboplastin time (aPTT) and pro-thrombin time (PT) tests are commonly used to assess the antithrombogenicity of the biomaterials in vitro [205,206]. The results obtained for the investigated wool–copper materials are presented in Figure 12 (aPTT) and Figure 13 (PT).

The aPTT results, which are the measure of the intrinsic, i.e., contact, coagulation mechanism of blood plasma through thrombin formation and fibrin clot polymerization, show that the clotting times of the modified samples were longer compared to the unmodified sample and the control sample. Moreover, as the amount of the metallic copper on the wool surface increased, the aPTT also increased. In the study presented by Kang et al., the researchers observed that the activation partial thromboplastin time (aPTT) slightly increased with the increase in the number of the grafted carboxyl groups on the surface of the polyurethane (PU) and poly(ethylene terephthalate) (PET) membranes. This result suggests a possible relationship between the presence of the carboxyl groups and the blood coagulation process [207,208]. Therefore, the prolonged aPTT time can potentially also be attributed to the presence of carboxyl groups that constitute the wool structure.

At the same time, it was observed that the presence of amino groups (-NH_2_) and carboxyl groups (-COOH) in wool did not have a significant impact on the PT (pro-thrombin time). Similarly, the surface concentration of metallic copper had no significant effect on the PT time. Therefore, it can be concluded that there was no disruption of the external factors of the blood coagulation pathway.

## 4. Conclusions

This paper investigated the biological potential of the new wool–copper materials (WO-Cu^(SpT)^(MBC)) obtained by the sputter deposition of copper on the fabric made from wool fibers. The following conclusions were obtained:

- The wool samples (WO) were successful modified by the surface deposition of metallic copper using a direct current (DC) magnetron sputtering system;
- The obtained results confirmed the antimicrobial protection of wool–copper materials (WO-Cu^(SpT)^(MBC)) against representative type of bacterial and fungal microorganisms according to EN ISO 20645:2006 and EN 14119:2003 standards [116,117];
- The investigated wool–copper materials have the ability to interact with bacterial DNA, resulting in breaks and changes in the conformation of the plasmid;
- The activated partial thromboplastin time (aPTT) of the modified samples (WO-Cu^(t)^(MBC)) was longer compared to the unmodified wool sample (WO). As the amount of the metallic copper on the wool surface increased, the aPTT also increased. No change was observed in the case of the pro-thrombin time;
- The good antimicrobial and antifungal effect of the wool–copper materials (WO-Cu^(SpT)^(MBC)) suggests a potential application as an antibacterial/antifungal material. Moreover, wool–copper (WO-Cu) materials may be applied as new customized materials, where the blood coagulation process could be well controlled by the copper concentration.

## Figures and Tables

**Table 1:** Characteristics of the textile material.

Parameter	
Surface mass	125 (±5) g/m^2^—determined in accordance with the standard *ISO 3801:1977* [113], Textiles. Woven fabrics. Determination of mass per unit length and mass per unit area. International Organization for Standardization: Geneva, Switzerland, 1977.
Textile material structure	Plain weave fabric.
The pH of the aqueous extract	7.5 (±0.5)—determined in accordance with the method described in the standard *ISO 3071:2020* [114], Textiles. Determination of the pH of aqueous extracts. International Organization for Standardization: Geneva, Switzerland, 2020.
The residue after dissolution in methane dichloride	0.5 (±0.1)%—determined in accordance with the method described in the standard *ISO 3074:2014* [115], Wool. Determination of dichloromethane-soluble matter in combed sliver. International Organization for Standardization: Geneva, Switzerland, 2014.
Thickness	0.40 (±0.5) mm—determined in accordance with the method described in the standard *ISO 5084:1996* [116], Textiles. Determination of thickness of textiles and textile products. International Organization for Standardization: Geneva, Switzerland, 1996.

**Table 2:** The sputtering procedure.

Method	Magnetron Sputtering
Equipment	DC magnetron sputtering system by P.P.H. Jolex s.c. (Czestochowa, Poland)
Target	Copper target of 99.99% purity from Testbourne Ltd. (Basingstoke, UK)
Distance between the target and substrate	15 cm
Deposition time	5, 10, and 15 min
Working atmosphere	Argon
Working pressure	2.4 × 10^−3^ mbar
Power discharge	0.5 kW
Power density	0.7 W/cm^2^

**Table 3:** Average amino acid composition of wool [121,122].

AA Abbr. (^a,b/^)	^a/^	Trp	His, Met	Ala, Ile, Lys, Phe, Tyr	Pro, Thr, Val	Gly	Asp ^c/^, Leu, Ser	Arg, Cys ^d/^	Glu ^e/^
	^b/^	W	H, M	A, I, K, F, Y	P, T, V	G	D, L, S	C, R	E
AA cont. [%]		0–0.5 ^f/^	0.6–0.9	3.5–5	5.8–6.5	4–8	7–8	7–10	13–16

Amino acids (AA) abbreviations: ^a,b/^ tri- or one-letter codes. AA cont. (%), amino acid percentage content in wool. ^c/^ Includes asparagine. ^d/^ Calculated as cysteine, cystine, and cysteic aid. ^e/^ Includes glutamine. ^f/^ Dependent on applied conditions of keratin degradation.

**Table 4:** Results of the determination of copper bulk content in WO-Cu samples.

Sample	Sp.T. ^a/^ (min.)	Copper Bulk Concentration		Sample Name ^e/^ WO-Cu^(SpT)^ (MBC)	Copper Bulk Concentration after Washing	
		(mg/kg) ^b,c/^	MBC ^d/^ (mol/kg)		(mg/kg) ^b,c/^	MBC ^d/^ (mol/kg)
WO	-	-	-	WO	-	-
WO-Cu^(5)^	5	3510	0.055	WO-Cu^(5)^(0.06)	2990	0.05
WO-Cu^(10)^	10	9020	0.14	WO-Cu^(10)^(0.14)	7960	0.13
WO-Cu^(15)^	15	24,270	0.38	WO-Cu^(15)^(0.38)	20,820	0.33

^a/^ Sp.T., sputtering deposition time (min). ^b/^ The results calculated from Equation (1). ^c/^ The results were measured in triplicate and are presented as a mean value with ± deviation equal to approximately 2%. ^d/^ MBC, copper molal bulk concentration (M_Cu_ = 63 550 mg/mol). ^e/^ WO-Cu^(SpT)^( MBC), wool fabric/copper material after corresponding sputtering deposition time (Sp.T.) with copper molal bulk concentration (MBC).

**Table 5:** EDS analysis of the chemical composition of the WO sample and WO-Cu samples.

Sample Name	Element Symbol	Element Name	Atomic Conc. (%)	Weight Conc. (%)
WO	C	Carbon	44.583	39.000
	N	Nitrogen	35.083	35.800
	O	Oxygen	19.049	22.200
	S	Sulfur	1.284	3.000
	Cu			
WO-Cu^(5)^(0.06)	C	Carbon	44.496	32.368
	N	Nitrogen	26.842	22.777
	O	Oxygen	21.029	20.380
	S	Sulfur	2.571	4.995
	Cu	Copper	5.062	19.481
WO-Cu^(10)^(0.14)	C	Carbon	53.169	36.300
	N	Nitrogen	13.937	11.100
	O	Oxygen	22.758	20.700
	S	Sulfur	2.633	4.800
	Cu	Copper	7.503	27.100
WO-Cu^(15)^(0.38)	C	Carbon	37.122	18.382
	N	Nitrogen	22.829	13.187
	O	Oxygen	17.264	11.389
	S	Sulfur	2.040	2.697
	Cu	Copper	20.745	54.346

Atomic Conc. (%), % as a function of the number of atoms; Weight Conc. (%), % as a function of weight of atoms.

**Table 6:** The results of the BET analysis of the investigated samples.

Sample Name	Specific Surface Area (SSA)	Total Pore Volume (TPV)
	m^2^/g	cm^3^/g
WO	0.2653	7.527 × 10^−4^
WO-Cu^(5)^(0.06)	0.1929	7.394 × 10^−4^
WO-Cu^(10)^(0.14)	0.1854	7.254 × 10^−4^
WO-Cu^(15)^(0.38)	0.1783	7.103 × 10^−4^

The results were measured in duplicate and are presented as a mean value with ± deviation equal to approximately 2%.

**Table 7:** Results of antimicrobial activity tests of WO-Cu^(SpT)^(MBC) materials.

POLYM-Cu(MBc) Material	Average Inhibition Zone (mm)				LIT.
	*Bacteria*		*Fungi*		
	*E. coli*	*S. aureus.*	*C. globosum*	*A. niger*	
WO	0 ^a/^				[111]
WO	0	0	0		This work ^b/^
WO-Cu(0.06)	1	1	1		
WO-Cu(0.14)	1	1	1		
WO-Cu(0.38)	3	2	1		
PET	0	0	0		[27]
PET-Cu(0.11)	1	1	3		
PET-Cu(0.22)	2	1	3		
PLA	0	0	0		[28]
PLA-Cu(0.16)	2	1	1		
PLA-Cu(0.43)	2	1	3		
PLA	0	0	0	0	[29]
PLA-ALG	0	0	0	0	
PLA-ALG-Cu^(+2)^(0.21)	3	2	3	3	
PLA-ALG-Cu^(+2)^(1.16)	3	4	3	3	

^a/^ The wool fabric showed antibacterial efficacy towards SA if interpreted according to the agar diffusion test as “no growth” under the textile sample [111]. ^b/^ Concentration of inoculum (CFU/mL): *E. coli*, 1.9 × 10^8^; *S. aureus*, 1.9 × 10^8^; *C. globosum*, 2.1 × 10^6^. Polymers: WO, wool fiber; PET, poly(ethylene terephthalate); PLA, polylactic acid; ALG, alginate; Cu(MBc), copper molal bulk concentration; PLA-ALG-Cu^(+2)^, PLA-ALG complex with copper sulfate.

**Table 8:** The literature’s in vitro antibacterial activity data of some human pathogenic bacteria by disc diffusion assay (ZOI, mm) of copper succinate (Cu(Succ)_2_) and copper nanoparticles (CuNPS).

CuNPS Synth. ^a–i/^	Pathogens								Ref.
	*E. coli*		*S. aureus*		*B. subtilis*		*C. albicans*		
	ZOI	Conc./Amount	ZOI	Conc./Amount	ZOI	Conc./Amount	ZOI	Conc./Amount	
CuSO_4_ → CuNPS ^a/^	11	25 μg/mL	9	25 μg/mL					[185]
Cu(OAc)_2_ → CuNPS ^b/^	10	25 μg/mL	10	25 μg/mL					[184]
			23	100 μg/mL					
Cu(OAc)_2_ → CuNPS ^c/^	2.6	0.21 mg/cm^2^	5.6	0.21 mg/cm^2^	2.8	0.21 mg/cm^2^	0	0.21 mg/cm^2^	[186]
Cu(Succ.)_2_ → CuNPS ^d/^	14	70 μL	10	70 μL					[187]
Cu(Succ.)_2_	30	70 μL	34	70 μL					
Cu(OAc)_2_ → CuNPS ^e/^	25	50 μL	21	50 μL			23	50 μL	[188]
WO→									
CuSO_4_ → CuO NPS ^f/^	4	0.28 M	4.2	0.28					[189]
	5	0.56 M	5.5	0.56 M					
CuCl_2_ → CuO NPS ^g/^	26	50 μL	21	50 μL			23	50 μL	[190]
WO → WO-CuSO_4_ → WO-CuNPS ^h,i/^ + WO-Cu_2_O NPS ^h,i/^			21	50 μL					[191]
			18	50 μL					

Copper derivatives: CuNPS, copper nanoparticles; CuO NPS, copper(II)oxide nanoparticles; Cu(OAc)_2_, copper acetate; Cu(Succ)_2_, copper succinate. CuNPS Syntheses: ^a/^ Bio-reduction using *Dryopteris manniana* extract (terpenoids, phenolics, alkaloids, flavonoids, steroids, saponins, and tannins present in the *D. manniana* leaf extracts serve as effective reducing agents); ^b/^ bio-reduction using *Piper nigrum* fruit extract (alkaloids, polyphenolic compounds, and terpenoids present in the *Piper nigrum* fruit extract serve as effective reducing agents and as stabilizing and capping agents); ^c/^ reduction by ascorbic acid; ^d/^ reduction by NaBH_4_; ^e/^ the reduction of copper acetate hydrate by modified polyol method; ^f/^ biosynthesis of copper (II) oxide nanoparticles (CuO NPs) using CuSO_4_, glucose, and *Halomonas elongate*; ^g/^ biosynthesis of copper (II) oxide nanoparticles (CuO NPs) using CuCl_2_ and apple peel extract under microwave (MW) irradiation; ^h,i/^ two-stage process consisting of (1) chelation of CuSO_4_ (0.5 M or 0.05 M) on wool and (2) subsequent reduction of CuSO_4_ complexed by means of ascorbic acid (Cu^2+^:Ascorbic acid = 0.5:0.6) ^h/^ or sodium borohydride (0.05 M:0.15 M) ^i/^.

## Data Availability

The data that support the findings of this study are available on request from the corresponding author.

## Supplementary Materials

The following supporting information can be downloaded at: https://www.mdpi.com/article/10.3390/ma17122878/s1, Table S1: Chemisorption of amino acids [127,128,129,130,131,132,133,134,135,136,137,138,139,140,141,142,143,144,145,146,147,148,149,150,151,152,153,154,155,156] and peptides [157,158,159,160,161,162,163,164,165] on copper phase.

## References

[1] Boulton A.J., Lewis C.T., Naumann D.N., Midwinter M.J. (2018). Prehospital Haemostatic Dressings for Trauma: A Systematic Review. Emerg. Med. J..

[2] Mohamed S.A., Hargest R. (2022). Surgical Anatomy of the Skin. Surg. Oxf..

[3] Sorg H., Tilkorn D.J., Hager S., Hauser J., Mirastschijski U. (2017). Skin Wound Healing: An Update on the Current Knowledge and Concepts. Eur. Surg. Res..

[4] Sorg H., Sorg C.G.G. (2023). Skin Wound Healing: Of Players, Patterns, and Processes. Eur. Surg. Res..

[5] D’Alessandro A., Anastasiadi A.T., Tzounakas V.L., Nemkov T., Reisz J.A., Kriebardis A.G., Zimring J.C., Spitalnik S.L., Busch M.P. (2023). Red Blood Cell Metabolism In Vivo and In Vitro. Metabolites.

[6] Dhivya S., Padma V.V., Santhini E. (2015). Wound Dressings—A Review. BioMedicine.

[7] Rezvani Ghomi E., Khalili S., Nouri Khorasani S., Esmaeely Neisiany R., Ramakrishna S. (2019). Wound Dressings: Current Advances and Future Directions. J. Appl. Polym. Sci..

[8] Michalicha A., Belcarz A., Giannakoudakis D.A., Staniszewska M., Barczak M. (2024). Designing Composite Stimuli-Responsive Hydrogels for Wound Healing Applications: The State-of-the-Art and Recent Discoveries. Materials.

[9] Wang H., Zhang L.-M. (2024). Intelligent Biobased Hydrogels for Diabetic Wound Healing: A Review. Chem. Eng. J..

[10] Yaron J.R., Gosangi M., Pallod S., Rege K. (2024). In Situ Light-activated Materials for Skin Wound Healing and Repair: A Narrative Review. Bioeng. Transl. Med..

[11] Zheng Q., Chen C., Liu Y., Gao J., Li L., Yin C., Yuan X. (2024). Metal Nanoparticles: Advanced and Promising Technology in Diabetic Wound Therapy. Int. J. Nanomed..

[12] Bishop A. (2023). Factors Influencing Dressing Choice in Wound Care: A Discussion. Br. J. Nurs..

[13] Choudhury A., Venkatesh D.N., Kumar P.J., Mohammed A.P.M. (2023). Advanced Wound Care with Biopolymers. Res. J. Pharm. Technol..

[14] Tran H.Q., Shahriar S.M.S., Yan Z., Xie J. (2023). Recent Advances in Functional Wound Dressings. Adv. Wound Care.

[15] Vivcharenko V., Trzaskowska M., Przekora A. (2023). Wound Dressing Modifications for Accelerated Healing of Infected Wounds. Int. J. Mol. Sci..

[16] Yousefian F., Hesari R., Jensen T., Obagi S., Rgeai A., Damiani G., Bunick C.G., Grada A. (2023). Antimicrobial Wound Dressings: A Concise Review for Clinicians. Antibiotics.

[17] Guo B., Dong R., Liang Y., Li M. (2021). Haemostatic Materials for Wound Healing Applications. Nat. Rev. Chem..

[18] Yu P., Zhong W. (2021). Hemostatic Materials in Wound Care. Burn. Trauma.

[19] Guo Y., Wang M., Liu Q., Liu G., Wang S., Li J. (2023). Recent Advances in the Medical Applications of Hemostatic Materials. Theranostics.

[20] Simões D., Miguel S.P., Ribeiro M.P., Coutinho P., Mendonça A.G., Correia I.J. (2018). Recent Advances on Antimicrobial Wound Dressing: A Review. Eur. J. Pharm. Biopharm..

[21] Nethi S.K., Das S., Patra C.R., Mukherjee S. (2019). Recent Advances in Inorganic Nanomaterials for Wound-Healing Applications. Biomater. Sci..

[22] Ijaola A.O., Akamo D.O., Damiri F., Akisin C.J., Bamidele E.A., Ajiboye E.G., Berrada M., Onyenokwe V.O., Yang S.-Y., Asmatulu E. (2022). Polymeric Biomaterials for Wound Healing Applications: A Comprehensive Review. J. Biomater. Sci. Polym. Ed..

[23] Mirhaj M., Labbaf S., Tavakoli M., Seifalian A. (2022). An Overview on the Recent Advances in the Treatment of Infected Wounds: Antibacterial Wound Dressings. Macromol. Biosci..

[24] Lu Z., Yu D., Nie F., Wang Y., Chong Y. (2023). Iron Nanoparticles Open Up New Directions for Promoting Healing in Chronic Wounds in the Context of Bacterial Infection. Pharmaceutics.

[25] Prete S., Dattilo M., Patitucci F., Pezzi G., Parisi O.I., Puoci F. (2023). Natural and Synthetic Polymeric Biomaterials for Application in Wound Management. J. Funct. Biomater..

[26] Verma D., Okhawilai M., Nangan S., Thakur V.K., Gopi S., Kuppusamy K., Sharma M., Uyama H. (2024). A Sustainable and Green Approach towards the Utilization of Biopolymers for Effective Wound Dressing Applications: A Detailed Review. Nano-Struct. Nano-Objects.

[27] Kudzin M.H., Kaczmarek A., Mrozińska Z., Olczyk J. (2020). Deposition of Copper on Polyester Knitwear Fibers by a Magnetron Sputtering System. Physical Properties and Evaluation of Antimicrobial Response of New Multi-Functional Composite Materials. Appl. Sci..

[28] Kudzin M.H., Mrozińska Z., Kaczmarek A., Lisiak-Kucińska A. (2020). Deposition of Copper on Poly(Lactide) Non-Woven Fabrics by Magnetron Sputtering—Fabrication of New Multi-Functional, Antimicrobial Composite Materials. Materials.

[29] Kudzin M.H., Boguń M., Mrozińska Z., Kaczmarek A. (2020). Physical Properties, Chemical Analysis, and Evaluation of Antimicrobial Response of New Polylactide/Alginate/Copper Composite Materials. Mar. Drugs.

[30] Kudzin M.H., Giełdowska M., Mrozińska Z., Boguń M. (2021). Poly(Lactic Acid)/Zinc/Alginate Complex Material: Preparation and Antimicrobial Properties. Antibiot. Basel Switz..

[31] Mrozińska Z., Ponczek M., Kaczmarek A., Boguń M., Sulak E., Kudzin M.H. (2023). Blood Coagulation Activities of Cotton–Alginate–Copper WO-Cu materials. Mar. Drugs.

[32] Mrozińska Z., Kudzin M.H., Ponczek M.B., Kaczmarek A., Król P., Lisiak-Kucińska A., Żyłła R., Walawska A. (2024). Biochemical Approach to Poly(Lactide)–Copper Composite—Impact on Blood Coagulation Processes. Materials.

[33] Konop M., Rybka M., Drapała A. (2021). Keratin Biomaterials in Skin Wound Healing, an Old Player in Modern Medicine: A Mini Review. Pharmaceutics.

[34] Ranjit E., Hamlet S., George R., Sharma A., Love R.M. (2022). Biofunctional Approaches of Wool-Based Keratin for Tissue Engineering. J. Sci. Adv. Mater. Devices.

[35] Ye W., Qin M., Qiu R., Li J. (2022). Keratin-Based Wound Dressings: From Waste to Wealth. Int. J. Biol. Macromol..

[36] Durrant P.J., Durrant B. (1962). Introduction to Advanced Inorganic Chemistry.

[37] Bertocci U., Wagman D.D., Bard A.J., Parsons R., Jordan J. (1985). Copper, silver and gold. Standard Potentials in Aqueous Solution.

[38] Allen S.E., Walvoord R.R., Padilla-Salinas R., Kozlowski M.C. (2013). Aerobic Copper-Catalyzed Organic Reactions. Chem. Rev..

[39] De Sousa P.V.F., De Oliveira A.F., Da Silva A.A., Lopes R.P. (2019). Environmental Remediation Processes by Zero Valence Copper: Reaction Mechanisms. Environ. Sci. Pollut. Res..

[40] Zerk T.J., Bernhardt P.V. (2018). Redox-Coupled Structural Changes in Copper Chemistry: Implications for Atom Transfer Catalysis. Coord. Chem. Rev..

[41] Borkow G., Gabbay J. (2005). Copper as a Biocidal Tool. Curr. Med. Chem..

[42] Borkow G., Gabbay J. (2009). Copper, An Ancient Remedy Returning to Fight Microbial, Fungal and Viral Infections. Curr. Chem. Biol..

[43] Grass G., Rensing C., Solioz M. (2011). Metallic Copper as an Antimicrobial Surface. Appl. Environ. Microbiol..

[44] Chyderiotis S., Legeay C., Verjat-Trannoy D., Le Gallou F., Astagneau P., Lepelletier D. (2015). Efficacy of Copper Surfaces in the Healthcare Environment: A Systematic Review. Antimicrob. Resist. Infect. Control.

[45] Vincent M., Duval R.E., Hartemann P., Engels-Deutsch M. (2018). Contact Killing and Antimicrobial Properties of Copper. J. Appl. Microbiol..

[46] Ermini M.L., Voliani V. (2021). Antimicrobial Nano-Agents: The Copper Age. ACS Nano.

[47] Crisan M.C., Teodora M., Lucian M. (2021). Copper Nanoparticles: Synthesis and Characterization, Physiology, Toxicity and Antimicrobial Applications. Appl. Sci..

[48] Maliki M., Ifijen I.H., Ikhuoria E.U., Jonathan E.M., Onaiwu G.E., Archibong U.D., Ighodaro A. (2022). Copper Nanoparticles and Their Oxides: Optical, Anticancer and Antibacterial Properties. Int. Nano Lett..

[49] Li X., Cong Y., Ovais M., Cardoso M.B., Hameed S., Chen R., Chen M., Wang L. (2023). Copper-based Nanoparticles against Microbial Infections. WIREs Nanomed. Nanobiotechnol..

[50] Yimeng S., Huilun X., Ziming L., Kejun L., Chaima M., Xiangyu Z., Yinchun H., Yan W., Di H. (2023). Copper-Based Nanoparticles as Antibacterial Agents. Eur. J. Inorg. Chem..

[51] Cortes A.A., Zuñiga J.M. (2020). The Use of Copper to Help Prevent Transmission of SARS-Coronavirus and Influenza Viruses. A General Review. Diagn. Microbiol. Infect. Dis..

[52] Jagaran K., Singh M. (2020). Nanomedicine for COVID-19: Potential of Copper Nanoparticles. Biointerface Res. Appl. Chem..

[53] Govind V., Bharadwaj S., Sai Ganesh M.R., Vishnu J., Shankar K.V., Shankar B., Rajesh R. (2021). Antiviral Properties of Copper and Its Alloys to Inactivate Covid-19 Virus: A Review. BioMetals.

[54] Lin N., Verma D., Saini N., Arbi R., Munir M., Jovic M., Turak A. (2021). Antiviral Nanoparticles for Sanitizing Surfaces: A Roadmap to Self-Sterilizing against COVID-19. Nano Today.

[55] Puchkova L.V., Kiseleva I.V., Polishchuk E.V., Broggini M., Ilyechova E.Y. (2021). The Crossroads between Host Copper Metabolism and Influenza Infection. Int. J. Mol. Sci..

[56] Rani I., Goyal A., Bhatnagar M., Manhas S., Goel P., Pal A., Prasad R. (2021). Potential Molecular Mechanisms of Zinc- and Copper-Mediated Antiviral Activity on COVID-19. Nutr. Res..

[57] Tortella G.R., Pieretti J.C., Rubilar O., Fernández-Baldo M., Benavides-Mendoza A., Diez M.C., Seabra A.B. (2022). Silver, Copper and Copper Oxide Nanoparticles in the Fight against Human Viruses: Progress and Perspectives. Crit. Rev. Biotechnol..

[58] Ramos-Zúñiga J., Bruna N., Pérez-Donoso J.M. (2023). Toxicity Mechanisms of Copper Nanoparticles and Copper Surfaces on Bacterial Cells and Viruses. Int. J. Mol. Sci..

[59] Zakharova O.V., Vasyukova I.A., Gusev A.A. (2023). Metal-Based Nanoparticles for the Diagnostics, Therapy, and Prevention of Viral Infections. Nanobiotechnol. Rep..

[60] Albalawi S.A., Albalawi R.A., Albalawi A.A., Alanazi R.F., Almahlawi R.M., Alhwity B.S., Alatawi B.D., Elsherbiny N., Alqifari S.F., Abdel-Maksoud M.S. (2024). The Possible Mechanisms of Cu and Zn in the Treatment and Prevention of HIV and COVID-19 Viral Infection. Biol. Trace Elem. Res..

[61] Gerwien F., Skrahina V., Kasper L., Hube B., Brunke S. (2018). Metals in Fungal Virulence. FEMS Microbiol. Rev..

[62] Robinson J.R., Isikhuemhen O.S., Anike F.N. (2021). Fungal–Metal Interactions: A Review of Toxicity and Homeostasis. J. Fungi.

[63] Amiri M.R., Alavi M., Taran M., Kahrizi D. (2022). Antibacterial, Antifungal, Antiviral, and Photocatalytic Activities of TiO _2_ Nanoparticles, Nanocomposites, and Bio-Nanocomposites: Recent Advances and Challenges. J. Public Health Res..

[64] Gurunathan S., Lee A.R., Kim J.H. (2022). Antifungal Effect of Nanoparticles against COVID-19 Linked Black Fungus: A Perspective on Biomedical Applications. Int. J. Mol. Sci..

[65] Pereira D., Carreira T.S., Alves N., Sousa Â., Valente J.F.A. (2022). Metallic Structures: Effective Agents to Fight Pathogenic Microorganisms. Int. J. Mol. Sci..

[66] Alselami A., Drummond R.A. (2023). How Metals Fuel Fungal Virulence, yet Promote Anti-Fungal Immunity. Dis. Model. Mech..

[67] Bellere A.D., Oh S., Zheng S., Fang M., Zuela E., Yi T.-H. (2023). Fungal Wonders: A Perspective on Various Fungal Benefits to Mankind. Food Res..

[68] Huang T., Li X., Maier M., O’Brien-Simpson N.M., Heath D.E., O’Connor A.J. (2023). Using Inorganic Nanoparticles to Fight Fungal Infections in the Antimicrobial Resistant Era. Acta Biomater..

[69] Madkhali O.A. (2023). A Comprehensive Review on Potential Applications of Metallic Nanoparticles as Antifungal Therapies to Combat Human Fungal Diseases. Saudi Pharm. J..

[70] Shinde B.H., Inamdar S.N., Nalawade S.A., Chaudhari S.B. (2023). A Systematic Review on Antifungal and Insecticidal Applications of Biosynthesized Metal Nanoparticles. Mater. Today Proc..

[71] Frezza M., Hindo S., Chen D., Davenport A., Schmitt S., Tomco D., Ping Dou Q. (2010). Novel Metals and Metal Complexes as Platforms for Cancer Therapy. Curr. Pharm. Des..

[72] Denoyer D., Masaldan S., La Fontaine S., Cater M.A. (2015). Targeting Copper in Cancer Therapy: ‘Copper That Cancer’. Metallomics.

[73] Shao S., Si J., Shen Y. (2019). Copper as the Target for Anticancer Nanomedicine. Adv. Ther..

[74] Lelièvre P., Sancey L., Coll J.-L., Deniaud A., Busser B. (2020). The Multifaceted Roles of Copper in Cancer: A Trace Metal Element with Dysregulated Metabolism, but Also a Target or a Bullet for Therapy. Cancers.

[75] Sharma M., Sharma A., Majumder S. (2020). Synthesis, Microbial Susceptibility and Anti-Cancerous Properties of Copper Oxide Nanoparticles- Review. Nano Express.

[76] Guan D., Zhao L., Shi X., Ma X., Chen Z. (2023). Copper in Cancer: From Pathogenesis to Therapy. Biomed. Pharmacother..

[77] Ji P., Wang P., Chen H., Xu Y., Ge J., Tian Z., Yan Z. (2023). Potential of Copper and Copper Compounds for Anticancer Applications. Pharmaceuticals.

[78] Wang X., Zhou M., Liu Y., Si Z. (2023). Cope with Copper: From Copper Linked Mechanisms to Copper-Based Clinical Cancer Therapies. Cancer Lett..

[79] Yang S., Song Y., Hu Y., Chen H., Yang D., Song X. (2023). Multifaceted Roles of Copper Ions in Anticancer Nanomedicine. Adv. Healthc. Mater..

[80] Yang Y., Li M., Chen G., Liu S., Guo H., Dong X., Wang K., Geng H., Jiang J., Li X. (2023). Dissecting Copper Biology and Cancer Treatment: ‘Activating Cuproptosis or Suppressing Cuproplasia’. Coord. Chem. Rev..

[81] Schuschke L.A., Saari J.T., Miller F.N., Schuschke D.A. (1995). Hemostatic Mechanisms in Marginally Copper-Deficient Rats. J. Lab. Clin. Med..

[82] Belozerskaya G.G., Makarov V.A., Zhidkov E.A., Malykhina L.S., Sergeeva O.A., Ter-Arutyunyants A.A., Makarova L.V. (2006). Local Hemostatics (A Review). Pharm. Chem. J..

[83] Ashfaq M., Verma N., Khan S. (2017). Highly Effective Cu/Zn-Carbon Micro/Nanofiber-Polymer Nanocomposite-Based Wound Dressing Biomaterial against the P. Aeruginosa Multi- and Extensively Drug-Resistant Strains. Mater. Sci. Eng. C.

[84] Saran M., Vyas S., Mathur M., Bagaria A. (2018). Green Synthesis and Characterisation of CuNPs: Insights into Their Potential Bioactivity. IET Nanobiotechnol..

[85] Van Rensburg M., Van Rooy M., Bester M., Serem J., Venter C., Oberholzer H. (2019). Oxidative and Haemostatic Effects of Copper, Manganese and Mercury, Alone and in Combination at Physiologically Relevant Levels: An Ex Vivo Study. Hum. Exp. Toxicol..

[86] Kong Y., Hou Z., Zhou L., Zhang P., Ouyang Y., Wang P., Chen Y., Luo X. (2021). Injectable Self-Healing Hydrogels Containing CuS Nanoparticles with Abilities of Hemostasis, Antibacterial Activity, and Promoting Wound Healing. ACS Biomater. Sci. Eng..

[87] Tarantino G., Citro V., Capone D., Gaudiano G., Sinatti G., Santini S.J., Balsano C. (2021). Copper Concentrations Are Prevalently Associated with Antithrombin III, but Also with Prothrombin Time and Fibrinogen in Patients with Liver Cirrhosis: A Cross-Sectional Retrospective Study. J. Trace Elem. Med. Biol..

[88] Alasvand N., Behnamghader A., Milan P.B., Simorgh S., Mobasheri A., Mozafari M. (2023). Tissue-Engineered Small-Diameter Vascular Grafts Containing Novel Copper-Doped Bioactive Glass Biomaterials to Promote Angiogenic Activity and Endothelial Regeneration. Mater. Today Bio.

[89] Liu G., Zu M., Wang L., Xu C., Zhang J., Reis R.L., Kundu S.C., Xiao B., Duan L., Yang X. (2024). CaO_2_–Cu_2_O Micromotors Accelerate Infected Wound Healing through Antibacterial Functions, Hemostasis, Improved Cell Migration, and Inflammatory Regulation. J. Mater. Chem. B.

[90] Wang M., Zhang W., Wang C., Xiao L., Yu L., Fan J. (2024). Hemostatic and Antibacterial Calcium–Copper Zeolite Gauze for Infected Wound Healing. RSC Adv..

[91] Harris E.D. (2004). A Requirement for Copper in Angiogenesis. Nutr. Rev..

[92] Finney L., Vogt S., Fukai T., Glesne D. (2009). Copper and angiogenesis: Unravelling a relationship key to cancer progression. Clin. Exp. Pharmacol. Physiol..

[93] D’Andrea L.D., Romanelli A., Di Stasi R., Pedone C. (2010). Bioinorganic Aspects of Angiogenesis. Dalton Trans..

[94] Saghiri M.A., Asatourian A., Orangi J., Sorenson C.M., Sheibani N. (2015). Functional Role of Inorganic Trace Elements in Angiogenesis—Part II: Cr, Si, Zn, Cu, and S. Crit. Rev. Oncol. Hematol..

[95] Devi S.R.B., Dhivya M.A., Sulochana K.N. (2016). Copper Transporters and Chaperones: Their Function on Angiogenesis and Cellular Signalling. J. Biosci..

[96] Jacobs A., Renaudin G., Forestier C., Nedelec J.-M., Descamps S. (2020). Biological Properties of Copper-Doped Biomaterials for Orthopedic Applications: A Review of Antibacterial, Angiogenic and Osteogenic Aspects. Acta Biomater..

[97] Xiao Y., Wang T., Song X., Yang D., Chu Q., Kang Y.J. (2020). Copper Promotion of Myocardial Regeneration. Exp. Biol. Med..

[98] Cucci L.M., Satriano C., Marzo T., La Mendola D. (2021). Angiogenin and Copper Crossing in Wound Healing. Int. J. Mol. Sci..

[99] Marzo T., La Mendola D. (2021). The Effects on Angiogenesis of Relevant Inorganic Chemotherapeutics. Curr. Top. Med. Chem..

[100] Šalandová M., Van Hengel I.A.J., Apachitei I., Zadpoor A.A., Van Der Eerden B.C.J., Fratila-Apachitei L.E. (2021). Inorganic Agents for Enhanced Angiogenesis of Orthopedic Biomaterials. Adv. Healthc. Mater..

[101] Lüthen F., Bergemann C., Bulnheim U., Prinz C., Neumann H.G., Podbielski A., Bader R., Rychly J. (2010). A Dual Role of Copper on the Surface of Bone Implants. Mater. Sci. Forum.

[102] Ryan E.J., Ryan A.J., González-Vázquez A., Philippart A., Ciraldo F.E., Hobbs C., Nicolosi V., Boccaccini A.R., Kearney C.J., O’Brien F.J. (2019). Collagen Scaffolds Functionalised with Copper-Eluting Bioactive Glass Reduce Infection and Enhance Osteogenesis and Angiogenesis Both in Vitro and in Vivo. Biomaterials.

[103] Eivazzadeh-Keihan R., Bahojb Noruzi E., Khanmohammadi Chenab K., Jafari A., Radinekiyan F., Hashemi S.M., Ahmadpour F., Behboudi A., Mosafer J., Mokhtarzadeh A. (2020). Metal-based Nanoparticles for Bone Tissue Engineering. J. Tissue Eng. Regen. Med..

[104] Bosch-Rué E., Diez-Tercero L., Giordano-Kelhoffer B., Delgado L.M., Bosch B.M., Hoyos-Nogués M., Mateos-Timoneda M.A., Tran P.A., Gil F.J., Perez R.A. (2021). Biological Roles and Delivery Strategies for Ions to Promote Osteogenic Induction. Front. Cell Dev. Biol..

[105] Ghosh S., Webster T.J. (2021). Metallic Nanoscaffolds as Osteogenic Promoters: Advances, Challenges and Scope. Metals.

[106] Pantulap U., Arango-Ospina M., Boccaccini A.R. (2022). Bioactive Glasses Incorporating Less-Common Ions to Improve Biological and Physical Properties. J. Mater. Sci. Mater. Med..

[107] Shen Q., Qi Y., Kong Y., Bao H., Wang Y., Dong A., Wu H., Xu Y. (2022). Advances in Copper-Based Biomaterials With Antibacterial and Osteogenic Properties for Bone Tissue Engineering. Front. Bioeng. Biotechnol..

[108] Shimabukuro M., Hayashi K., Kishida R., Tsuchiya A., Ishikawa K. (2022). Surface Functionalization with Copper Endows Carbonate Apatite Honeycomb Scaffold with Antibacterial, Proangiogenic, and pro-Osteogenic Activities. Biomater. Adv..

[109] Amantay M., Ma Y., Chen L., Osman H., Mengping L., Jiang T., Zhou T., Ye T., Wang Y. (2023). Electrospinning Fibers Modified with Near Infrared Light-Excited Copper Nanoparticles for Antibacterial and Bone Regeneration. Adv. Mater. Interfaces.

[110] Heliopoulos N.S., Papageorgiou S.K., Galeou A., Favvas E.P., Katsaros F.K., Stamatakis K. (2013). Effect of Copper and Copper Alginate Treatment on Wool Fabric. Study of Textile and Antibacterial Properties. Surf. Coat. Technol..

[111] Ivankovic T., Rajic A., Ercegovic Razic S., Rolland Du Roscoat S., Skenderi Z. (2022). Antibacterial Properties of Non-Modified Wool, Determined and Discussed in Relation to ISO 20645:2004 Standard. Molecules.

[112] Ruberto M.F., Marongiu F., Barcellona D., Favaloro E.J., Gosselin R.C. (2023). Performance and Interpretation of Clot Waveform Analysis. Hemostasis and Thrombosis.

[113] (1977). Textiles—Woven Fabrics—Determination of Mass per Unit Length and Mass per Unit Area.

[114] (2020). Textiles—Determination of pH of Aqueous Extract.

[115] (2014). Wool—Determination of Dichloromethane-Soluble Matter in Combed Sliver.

[116] (1996). Textiles—Determination of Thickness of Textiles and Textile Products.

[117] Analytical Methods for Atomic Absorption Spectroscopy. The Perkin-Elmer Corporation, 1996, p. 41. www.lasalle.edu/.

[118] (2010). Textiles-Tests for Color Fastness—Part C06: Color Fastness to Domestic and Commercial Laundering.

[119] (2021). Microbeam Analysis—Selected Instrumental Performance Parameters for the Specification and Checking of Energy-Dispersive X-ray Spectrometers (EDS) for Use with a Scanning Electron Microscope (SEM) or an Electron Probe Microanalyser (EPMA).

[120] (2006). Textile Fabrics—Determination of Antibacterial Activity—Agar Diffusion Plate Test.

[121] (2003). Testing of Textiles. Evaluation of the Action of Microfungi. Visual Method.

[122] Juszczak M., Das S., Kosińska A., Rybarczyk-Pirek A.J., Wzgarda-Raj K., Tokarz P., Vasudevan S., Chworos A., Woźniak K., Rudolf B. (2023). Piano-Stool Ruthenium(II) Complexes with Maleimide and Phosphine or Phosphite Ligands: Synthesis and Activity against Normal and Cancer Cells. Dalton Trans..

[123] Zhang N., Wang Q., Yuan J., Cui L., Wang P., Yu Y., Fan X. (2018). Highly Efficient and Eco-Friendly Wool Degradation by L-Cysteine-Assisted Esperase. J. Clean. Prod..

[124] Rippon J.A., Christoe J.R., Denning R.J., Evans D.J., Huson M.G., Lamb P.R., Millington K.R., Pierlot A.P., Mark H.F. (2016). Wool: Structure, Properties, and Processing. Encyclopedia of Polymer Science and Technology.

[125] Giteru S.G., Ramsey D.H., Hou Y., Cong L., Mohan A., Bekhit A.E.A. (2023). Wool Keratin as a Novel Alternative Protein: A Comprehensive Review of Extraction, Purification, Nutrition, Safety, and Food Applications. Compr. Rev. Food Sci. Food Saf..

[126] Shorland F.B., Gray J.M. (1970). The Preparation of Nutritious Protein from Wool. Br. J. Nutr..

[127] Zhao X., Wang H., Zhao R.G., Yang W.S. (2001). Self-assembly of amino acids on the Cu(001) surface. Mater. Sci. Eng. C.

[128] Rankin R.B., Sholl D.S. (2005). Structures of glycine, enantiopure alanine, and racemic alanine adlayers; on Cu(110) and Cu(100) surfaces. J. Phys. Chem. B.

[129] Cheong W.Y., Huang Y., Dangaria N., Gellman A.J. (2010). Probing enantioselectivity on chirally modified Cu(110), Cu(100), and Cu(111) surfaces. Langmuir.

[130] Gladys M.J., Stevens A.V., Scott N.R., Glenn J., Batchelor D., Held G. (2007). Enantiospecific adsorption of alanine on the chiral Cu(531) surface. J. Phys. Chem. C.

[131] Thomsen L., Wharmby M.T., Riley D.P., Held G., Gladys M.J. (2009). The adsorption and stability of sulfur containing amino acids on Cu(531). Surf. Sci..

[132] Ge S.P., Lu C., Zhao R.G., Chen Q. (2006). Scanning tunneling microscopy investigation of leucine and asparagine adsorbed on Cu(111). Appl. Surf. Sci..

[133] Karagoz B., Reinicker A., Gellman A.J. (2019). Kinetics and mechanism of aspartic acid adsorption and its explosive decomposition on Cu(100). Langmuir.

[134] Totani R., Méthivier C., Cruguel H., Pradier C.M., Humblot V. (2017). Deciphering the adsorption mechanisms of RGD subunits: L-Aspartic acid on Cu(110). J. Phys. Chem. C.

[135] Cramer L.A., Larson A., Daniels A.S., Sykes E.C.H., Gellman A.J. (2023). Molecular origins of chiral amplification on an achiral surface: 2D monolayers of aspartic acid on Cu(111). ACS Nano.

[136] Kim J.W., Lee Y.M., Lee S.M., Son M.J., Kang H., Park Y. (2010). Surface reaction of sulfur-containing amino acids on Cu(110). Langmuir.

[137] Kumar D., Jain N., Jain V., Rai B. (2020). Amino acids as copper corrosion inhibitors: A density functional theory approach. App. Surf. Sci..

[138] Wang C., Luo X., Jia Z. (2017). Linkage, charge state and layer of l-cysteine on copper surfaces. Colloids Surf. B Biointerfaces.

[139] Rodríguez-Zamora P., Cordero-Silis C.A., Fabila J., Luque-Ceballos J.C., Buendía B., Heredia-Barbero A., Garzón I.L. (2022). Interaction mechanisms and interface configuration of cysteine adsorbed on gold, silver, and copper nanoparticles. Langmuir.

[140] Marti E.M., Methivier C., Dubot P., Pradier C.M. (2003). Adsorption of (S)-histidine on Cu(110) and oxygen-covered Cu(110), a combined Fourier transform reflection absorption infrared spectroscopy and force field calculation study. J. Phys. Chem. B.

[141] Bouri M., Lekka C. (2023). L-Glutamine coating on antibacterial cu surface by density functional theory. Crystals.

[142] Barlow S.M., Kitching K.J., Haq S., Richardson N.V. (1998). A study of glycine adsorption on a Cu(110) surface using reflection absorption infrared spectroscopy. Surf. Sci..

[143] Booth N.A., Woodruff D.P., Schaff O., Gießel T., Lindsay R., Baumgärtel P., Bradshaw A.M. (1998). Determination of the local structure of glycine adsorbed on Cu(110). Surf. Sci..

[144] Hasselström J., Karis O., Weinelt M., Wassdahl N., Nilsson A., Nyberg M., Pettersson L.G.M., Samant M.G., Stöhr J. (1998). The adsorption structure of glycine adsorbed on Cu(110); comparison with formate and acetate/Cu(110). Surf. Sci..

[145] Carravetta V., Monti S., Li C., Ågren H. (2013). Theoretical simulations of structure and X-ray photoelectron spectra of glycine and diglycine adsorbed on Cu(110). Langmuir.

[146] Zhao X., Zhao R.G., Yang W.S. (2000). Scanning tunneling microscopy investigation of L-lysine adsorbed on Cu(001). Langmuir.

[147] Humblot V., Méthivier C., Raval R., Pradier C.-M. (2007). Amino acid and peptides on Cu(110) surfaces: Chemical and structural analyses of L-lysine. Surf. Sci..

[148] Tielens F., Humblot V., Pradier C.-M. (2008). Elucidation of the low coverage chiral adsorption assembly of l-lysine on Cu(110) surface: A theoretical study. Surf. Sci..

[149] Eralp T., Shavorskiy A., Held G. (2011). The adsorption geometry and chemical state of lysine on Cu(110). Surf. Sci..

[150] Méthivier C., Humblot V., Pradier C.-M. (2015). L-Methionine adsorption on Cu(110), binding and geometry of the amino acid as a function of coverage. Surf. Sci..

[151] Ghiringhelli L.M., Delle Site L. (2008). Phenylalanine near inorganic surfaces: Conformational statistics vs specific chemistry. J. Amer. Chem. Soc..

[152] Eralp T., Shavorskiy A., Zheleva Z.V., Held G., Kalashnyk N., Ning Y., Linderoth T.R. (2010). Global and local expression of chirality in serine on the Cu(110) surface. Langmuir.

[153] Eralp T., Ievins A., Shavorskiy A., Jenkins S.J., Held G. (2012). The importance of attractive three-point interaction in enantioselective surface chemistry: Stereospecific adsorption of serine on the intrinsically chiral Cu(531) surface. J. Amer. Chem. Soc..

[154] Lee H., Kim H.S. (2020). Confirmation of initial stable adsorption structures of leucine and tyrosine adsorbed on a Cu(110) surface. Appl. Sci..

[155] Zhao X., Zhao R.G., Yang W.S. (2002). Self-assembly of L-tryptophan on the Cu(001) surface. Langmuir.

[156] Wang D., Xu Q.-M., Wan L.-J., Bai C.-L., Jin G. (2003). Adsorption of enantiomeric and racemic tyrosine on Cu(111): A scanning tunneling microscopy study. Langmuir.

[157] Feyer V., Plekan O., Tsud N., Lyamayev V., Cháb V., Matolín V., Prince K.C., Carravetta V. (2010). Adsorption structure of glycyl-glycine on Cu(110). J. Phys. Chem. C.

[158] Monti S., Carravetta V., Li C., Ågren H. (2014). A computational study of the adsorption and reactive dynamics of diglycine on Cu(110). J. Phys. Chem. C.

[159] Methivier C., Humblot V., Pradier C.-M. (2016). UHV deposition of the Gly-Pro dipeptide on Cu(110) by sublimation or electrospray ionization. J. Phys. Chem. C.

[160] Stensgaard I. (2003). Adsorption of di-L-alanine on Cu(110) investigated with scanning tunneling microscopy. Surface Sci..

[161] Tomba G., Lingenfelder M., Costantini G., Kern K., Klappenberger F., Barth J.V., Ciacchi L.C., De Vita A. (2007). Structure and energetics of diphenylalanine self-assembling on Cu(110)+. J. Phys. Chem. A.

[162] Mervinetsky E., Alshanski I., Hamo Y., Sandonas L.M., Dianat A., Buchwald J., Gutierrez R., Cuniberti G., Hurevich M., Yitzchaik S. (2017). Copper induced conformational changes of tripeptide monolayer based impedimetric biosensor. Biosensor. Sci. Rep..

[163] Gellini C., Sabatino G., Papini A.M., Muniz-Miranda M. (2014). SERS study of a tetrapeptide based on histidine and glycine residues, adsorbed on copper/silver colloidal nanoparticles. J. Raman Spectrosc..

[164] Mauri E., Sacchetti A., Rossi F. (2016). The synthesis of RGD-functionalized hydrogels as a tool for therapeutic applications. J. Vis. Exp..

[165] Méthivier C., Cornette P., Costa D., Landoulsi J. (2023). Electrospray ion beam deposition of small peptides on solid surfaces: A molecular level description of the glutathione/copper interface. Appl. Surface Sci..

[166] Kobayashi Y., Abe Y., Maeda T., Yasuda Y., Morita T. (2014). A metal–metal bonding process using metallic copper nanoparticles produced by reduction of copper oxide nanoparticles. J. Mater. Res. Technol..

[167] Gond-Charton P., Imbert B., Benaissa L., Carron V., Verdier M. (2015). Kinetics of low temperature direct copper–copper bonding. Microsyst. Technol..

[168] Lepetit C., Fau P., Fajerwerg K., Kahn M.L., Silvi B. (2017). Topological analysis of the metal-metal bond: A tutorial review. Coord. Chem. Rev..

[169] Mou Y., Peng Y., Zhang Y., Cheng H., Chen M. (2018). Cu-Cu bonding enhancement at low temperature by using carboxylic acid surface-modified Cu nanoparticles. Mater. Lett..

[170] Wilson R.J., Lichtenberger N., Weinert B., Dehnen S. (2019). Intermetalloid and heterometallic clusters combining p-block (semi)metals with d- or f-block metals. Chem. Rev..

[171] Lin P.F., Tran D.P., Liu H.C., Li Y.Y., Chen C. (2022). Interfacial characterization of low-temperature Cu-to-Cu direct bonding with chemical mechanical planarized nanotwinned Cu films. Materials.

[172] Arai S., Nakajima S., Shimizu M., Shimizu M., Aizawa M., Kiyoshi O. (2023). Direct Cu-Cu bonding by low-temperature sintering using three-dimensional nanostructured plated Cu films. Mater. Today Commun..

[173] Yao D., Wang Y., Li Y., Li A., Zhen Z., Lv J., Sun F., Yang R., Luo J., Jiang Z. (2023). Scalable synthesis of Cu clusters for remarkable selectivity control of intermediates in consecutive hydrogenation. Nat. Commun..

[174] Paul S., Hewitt A., Rana S., Goswami P. (2023). Development of novel parameters for characterising scale morphology of wool fibre and its correlation with dye diffusion coefficient of acid dye. Sci. Rep..

[175] Xu W., Ke G., Wu J., Wang X. (2006). Modification of wool fiber using steam explosion. Eur. Polym. J..

[176] Essaket M., Wazna M.E., Boukhriss A., Essaket I., Bouari A.E., Cherkaoui O., Maliki A.E. (2023). A Comparative Study and Thermophysical Characterization of Wool Fiber from Different Regions of Morocco. Biomass Convers. Biorefinery.

[177] Bläker C., Muthmann J., Pasel C., Bathen D. (2019). Characterization of Activated Carbon Adsorbents—State of the Art and Novel Approaches. ChemBioEng Rev..

[178] Airaksinen S. (2005). Role of Excipients in Moisture Sorption and Physical Stability of Solid Pharmaceutical Formulations. Ph.D. Thesis.

[179] Thommes M., Kaneko K., Neimark A.V., Olivier J.P., Rodriguez-Reinoso F., Rouquerol J., Sing K.S.W. (2015). Physisorption of Gases, with Special Reference to the Evaluation of Surface Area and Pore Size Distribution (IUPAC Technical Report). Pure Appl. Chem..

[180] Qi L., Tang X., Wang Z., Peng X. (2017). Pore Characterization of Different Types of Coal from Coal and Gas Outburst Disaster Sites Using Low Temperature Nitrogen Adsorption Approach. Int. J. Min. Sci. Technol..

[181] Boinovich L.B., Kaminsky V.V., Domantovsky A.G., Emelyanenko K.A., Aleshkin A.V., Zulkarneev E.R., Kiseleva I.A., Emelyanenko A.M. (2019). Bactericidal activity of superhydrophobic and superhydrophilic copper in bacterial dispersions. Langmuir.

[182] Chang T., Prasath Babu R., Zhao C.W., Johnson M., Hedström P., Odnevall I., Leygraf C. (2021). High-resolution microscopical studies of contact killing mechanisms on copper-based surfaces. ACS Appl. Mater. Interfaces.

[183] Hans M., Mathews S., Mucklich F., Solioza M. (2016). Physicochemical properties of copper important for its antibacterial activity and development of a unified model. Biointerphases.

[184] Kiranmayee M., Rajesh N., Vidya Vani M., Khadri H., Suresh A.M., Chinni V., Ramachawolran G., Riazunnisa K., Moussa A.Y. (2023). Green synthesis of Piper nigrum copper-based nanoparticles: In silico study and ADMET analysis to assess their antioxidant, antibacterial, and cytotoxic effects. Front. Chem..

[185] Ejidike I.P., Ijimdiya R.U., Emmanuel-Akerele H.A., Emmanuel G.C., Ejidike O.M., Bamigboye M.O., Seyinde D.O., Olaleru A., Tanimowo W.O., Awolope R.O. (2023). Biosynthesis, characterization, and antimicrobial assessment of metal nanoparticles from Dryopteris Manniana (Hook.) C. Chr leaf extract. Bull. Pharmac. Sci. Assiut.

[186] Raspolli Galletti A.M., Antonetti C., Marracci M., Piccinelli F., Tellini B. (2013). Novel microwave-synthesis of Cu nanoparticles in the absence of any stabilizing agent and their antibacterial and antistatic applications. Appl. Surf. Sci..

[187] Karthik A.D., Geetha K. (2013). Synthesis of copper precursor, copper and its oxide nanoparticles by green chemical reduction method and its antimicrobial activity. J. Appl. Pharmac. Sci..

[188] Rad M., Taran M., Alavi M. (2018). Effect of incubation time, CuSO4 and glucose concentrations on biosynthesis of copper oxide (CuO) nanoparticles with rectangular shape and antibacterial activity: Taguchi method approach. Nano Biomed. Eng..

[189] Ramyadevi J., Jeyasubramanian K., Marikani A., Rajakumar G., Rahuman A.A. (2012). Synthesis and antimicrobial activity of copper nanoparticles. Mater. Lett..

[190] Rajamohan R., Raorane C.J., Kim S.-C., Ashokkumar S., Lee Y.R. (2023). Novel Microwave synthesis of copper oxide nanoparticles and appraisal of the antibacterial application. Micromachines.

[191] Ivanauskas R., Ancutiene I., Milašienė D., Ivanauskas A., Bronusiene A. (2022). Effect of reducing agent on characteristics and antibacterial activity of copper-containing particles in textile materials. Materials.

[192] Zhao J., Yang G., Zhang C., Zhang Y., Zhang S., Zhang P. (2019). Synthesis of water-soluble Cu nanoparticles and evaluation of their tribological properties and thermal conductivity as a water-based additive. Friction.

[193] Song Y., Xie X., Liu Y., Zhu Z., Sun L. (2023). Nanoscale Study of DNA–Cu^2+^ Interactions by Liquid-Cell Electron Microscopy. ACS Omega.

[194] Erxleben A. (2018). Interactions of Copper Complexes with Nucleic Acids. Coord. Chem. Rev..

[195] De Souza Í.P., Machado B.D.P., De Carvalho A.B., Binatti I., Krambrock K., Molphy Z., Kellett A., Pereira-Maia E.C., Silva-Caldeira P.P. (2019). Exploring the DNA Binding, Oxidative Cleavage, and Cytotoxic Properties of New Ternary Copper(II) Compounds Containing 4-Aminoantipyrine and N,N-Heterocyclic Co-Ligands. J. Mol. Struct..

[196] Pham A.N., Xing G., Miller C.J., Waite T.D. (2013). Fenton-like Copper Redox Chemistry Revisited: Hydrogen Peroxide and Superoxide Mediation of Copper-Catalyzed Oxidant Production. J. Catal..

[197] Freudenthal B.D. (2017). Base Excision Repair of Oxidative DNA Damage from Mechanism to Disease. Front. Biosci..

[198] Shim S.-Y., Kim H.-S. (2013). Oxidative Stress and the Antioxidant Enzyme System in the Developing Brain. Korean J. Pediatr..

[199] Chen X., Chen J., Huang N. (2022). The Structure, Formation, and Effect of Plasma Protein Layer on the Blood Contact Materials: A Review. Biosurf. Biotribol..

[200] Hanson S.R., Tucker E.I., Latour R.A., Wagner W.R., Sakiyama-Elbert S.E., Zhang G., Yaszemski M.J. (2020). 2.2.6—Blood Coagulation and Blood–Material Interactions. Biomaterials Science.

[201] Vogler E.A., Siedlecki C.A. (2009). Contact Activation of Blood-Plasma Coagulation. Biomaterials.

[202] Liu Y., Zhang Y., Yao W., Chen P., Cao Y., Shan M., Yu S., Zhang L., Bao B., Cheng F.-F. (2024). Recent Advances in Topical Hemostatic Materials. ACS Appl. Bio Mater..

[203] Qin H., Sun C., He C., Wang D., Cheng C., Nie S., Sun S., Zhao C. (2014). High Efficient Protocol for the Modification of Polyethersulfone Membranes with Anticoagulant and Antifouling Properties via in Situ Cross-Linked Copolymerization. J. Membr. Sci..

[204] Liu Y., Li G., Han Q., Lin H., Li Q., Hua J., Liu F. (2020). Anticoagulant Dialyzer with Enhanced Ca2+ Chelation and Hydrophilicity for Heparin Free Hemodialysis. J. Membr. Sci..

[205] Silver B.J., Lichtin A., Bartholomew J.R. (2014). Prolongation of Both PT and APTT. The Coagulation Consult: A Case-Based Guide.

[206] Yang R., Zubair M., Moosavi L. (2024). Prothrombin Time. StatPearls.

[207] Kim Y.J., Kang I.-K., Huh M.W., Yoon S.-C. (2000). Surface Characterization and in Vitro Blood Compatibility of Poly(Ethylene Terephthalate) Immobilized with Insulin and/or Heparin Using Plasma Glow Discharge. Biomaterials.

[208] Kang I.-K., Kwon O.H., Kim M.K., Lee Y.M., Sung Y.K. (1997). In Vitro Blood Compatibility of Functional Group-Grafted and Heparin-Immobilized Polyurethanes Prepared by Plasma Glow Discharge. Biomaterials.

