# Supplementary material for: Biochemical Behavior, Influence on Cell DNA Condition, and Microbiological Properties of Wool and Wool–Copper Materials
Source: Materials (Basel). 2024 Jun 12;17(12):2878. doi: 10.3390/ma17122878 (PMC11204859; doi:10.3390/ma17122878)
Supplement: Supplementary file 1 [file materials-17-02878-s001.zip › materials-2978640-supplementary.pdf]

materials

MDPI

Article

Biochemical Behavior, Influence on Cell DNA Condition, and Microbiological Properties of Wool and Wool–Copper Materials

Zdzisława Mrozińska [1], Anna Kaczmarek [1], Małgorzata Świerczyńska [1,2], Michał Juszczak [1,3], and Marcin H. Kudzin [1,*]

[1] Łukasiewicz Research Network—Lodz Institute of Technology, 19/27 Marii Sklodowskiej-Curie Str., 90-570 Lodz, Poland; zdzislawa.mrozinska@lit.lukasiewicz.gov.pl (Z.M.); anna.kaczmarek@lit.lukasiewicz.gov.pl (A.K.); malgorzata.swierczynska@lit.lukasiewicz.gov.pl (M.Ś.); michal.juszczak@lit.lukasiewicz.gov.pl (M.J.)

[2] Institute of Polymer and Dye Technology, Faculty of Chemistry, Lodz University of Technology, Stefanowskiego 16, 90-537 Lodz, Poland

[3] Department of Molecular Genetics, Faculty of Biology and Environmental Protection, University of Lodz, 90-236 Lodz, Poland

* Correspondence: marcin.kudzin@lit.lukasiewicz.gov.pl; Tel.: +48-42-6163121

Table S1. Chemisorption of amino acids [127–156] and peptides [157–165] on copper phase.

Amino Acid/ peptide	Copper Phase						
	**Cu[001]**	**Cu[100]**	**Cu[110]**	**Cu[111]**	**Cu{531}**	**Cu-Electr.**	**CuNPS**
Ala	[127]	[128; 129]	[130]		[131; 132]		
Asn	[127]			[133]			
Asp	[127]	[134]	[135]	[136]			
Cys			[137]	[138]	[139]	[140]	[141]
Cys-Cys			[136; 141]				
Gln				[142]			
Glu				[137]			
Gly	[127]	[128]	[143-146]	[137]			
His			[141]				
Lys	[127; 147]		[148-150]				
Met			[136; 151]	[137]	[131]		
Phe	[127]			[152]			
Ser			[153]		[154]		
Trp	[155]						
Tyr			[154]	[156]			
Gly-Gly			[145; 157; 158]				
Gly-Pro			[159]				
Ala-Ala			[160]				
Phe-Phe			[161]				
Gly-Gly-His							[162]
Glu-Cys-Gly			[163]	[163]			
Ac-His-Gly-His-Gly							[164][a]
$(Gly)_2$-Lys-$(Gly)_2$-Lys-$(Gly)_2$-$(His)_5$							[165][a]

Cu(xyz) – copper crystals; Cu-Electr. Copper electrode phase; CuNPS – copper nano particle; [a]/Cu colloid
